# Curcumin and its Analogs and Carriers: Potential Therapeutic Strategies for Human Osteosarcoma

**DOI:** 10.7150/ijbs.80590

**Published:** 2023-02-13

**Authors:** Ko-Hsiu Lu, Peace Wun-Ang Lu, Eric Wun-Hao Lu, Chiao-Wen Lin, Shun-Fa Yang

**Affiliations:** 1 Department of Orthopedics, Chung Shan Medical University Hospital, Taichung, Taiwan; 2 School of Medicine, Chung Shan Medical University, Taichung, Taiwan; 3 Morrison Academy Taichung, Taichung, Taiwan; 4 Department of Mechanical Engineering, College of Engineering, University of Michigan, Ann Arbor, MI, USA; 5 Institute of Oral Sciences, Chung Shan Medical University, Taichung, Taiwan; 6 Institute of Medicine, Chung Shan Medical University, Taichung, Taiwan; 7 Department of Medical Research, Chung Shan Medical University Hospital, Taichung, Taiwan

**Keywords:** Bioavailability, carrier, curcumin analogs, therapeutic efficacy, human osteosarcoma

## Abstract

Curcumin is a natural polyphenol phytochemical derived from turmeric with antioxidant, anti-inflammatory, and anticancer properties but is concerned about poor solubility in water, absorption, and metabolic stability. Potent metastatic osteosarcoma is the most common primary bone cancer in children, adolescents, and young adults. It is responsible for low survival rates because of its high rate of metastasis to the lungs. To improve poor bioavailability, numerous curcumin analogs were developed to possess anticancer characteristics through a variety of biological pathways involved in cytotoxicity, proliferation, autophagy, sensitizing chemotherapy, and metastases. This review provides an overview of their various pharmacological functions, molecular mechanisms, and therapeutic potential as a remedy for human osteosarcoma. To enhance therapeutic efficacy, several liposomal nanoparticles, nanocarriers, multifunctional micelles, and three-dimensional printed scaffolds have also been developed for the controlled delivery of curcumin targeting human osteosarcoma cells. Consequently, curcumin and several potential analogs and delivery formulations are optimistic candidates to improve the currently available strategy for human osteosarcoma. However, further insight into the mechanism of action of promising curcumin analogs and the development of carriers in clinical trials of osteosarcoma needs to be investigated to improve their overall potency and clinical utility, in particular the anti-metastatic effect.

## 1. Introduction

Turmeric (curry powder), a cooking spice, a food preservative, and a natural yellow food coloring in many foods, such as curries and mustards, as well as a cosmetic agent for skin care in Asia, Europe, and America, has been integral to traditional medicinal systems for centuries, such as Indian Ayurveda 1 and Chinese herbs 2. For thousands of years, turmeric has been used to treat common colds, fevers, stomachaches, liver diseases, open wounds, skin diseases, and chronic inflammations. After epidemiological studies, turmeric may have the ability to lower cancer incidence rates by 10-50% among those who regularly consume the spice 3.

**Figure 1:**
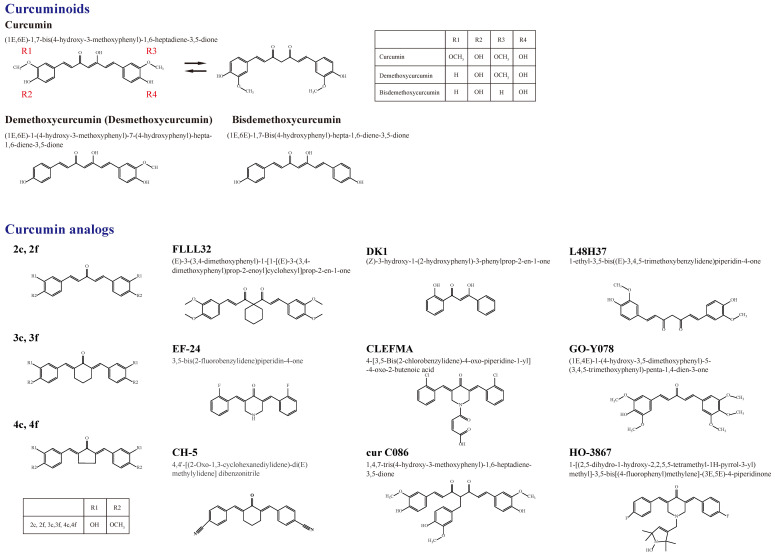
A summary of the chemical structures of curcumin and its natural (demethoxycurcumin and bisdemethoxycurcumin) and current synthetic analogues (2c ,2f , 3c ,3f , 4c, 4f, FLLL32, EF-24, CH-5, DK1, CLEFMA, cur C086, L48H37, GO-Y078, and HO-3867) developed to overcome the limitations of curcumin and its involvement in the molecular impact on human osteosarcoma cells.

Owing to 2-9% curcuminoids, comprising curcumin (approximately 75-80%), demethoxycurcumin (desmethoxycurcumin, DMC) (approximately 15-20%), bisdemethoxycurcumin (BDMC) (approximately 3-5%), and a minor component of cyclic curcumin, turmeric displays the yellow color characteristic 4, 5 (Figure 1). Curcumin (diferuloylmethane), the main curcuminoid, was first discovered from the turmeric rhizome of the East Indian plant *Curcuma longa* Linn. by two German scientists Vogel and Pelletier in 1815 5, 6. It is a member of the Zingiberaceae (ginger) family of perennial botanicals with a long history of traditional uses that range from folk medicine to various culinary preparations 7.

## 2. Curcumin

### 2.1 Introducing the structure

The chemical structure of curcumin, (1E,6E)-1,7-bis(4-hydroxy-3-methoxyphenyl)-1,6-heptadiene-3,5-dione with a molecular weight of 368.4 g/mol and chemical formula C_21_H_20_O_6_, is a lipid-soluble polyphenolic phytochemical, which comprises two aryl buten-2-one (feruloyl) chromophores joined by a methylene group 8, 9. The phenolic compound curcumin, approximately 2-5% of the Indian spice turmeric, was first described by Lampe and Milobedeska in 1913, and its biological properties were described as responsible for most of the therapeutic efficacy of turmeric in the 1970s 10-13. After being recognized for its ability to suppress the activation of the transcription factor nuclear factor κ-light-chain enhancer of activated B cell (NF-κB), curcumin has been used as an anticancer agent since the 1990s 14.

### 2.2 Molecular mechanism and pharmacology of curcumin

Considered pharmacologically safe, curcumin possesses various effects, including antioxidant 15, anti-inflammatory 13, and anti-septic properties 16, and also modifies its influence on the expression of genes involved in cytotoxicity, angiogenesis, metastasis, and drug resistance 17, 18. Curcumin mediates its promising activities by regulating several key molecular targets. They include (1) transcription factors [e.g., activator protein (AP)-1, β-catenin, NF-κB, peroxisome proliferator-activated receptor (PPAR)-γ, and signal transducer and activator of transcription (STAT)] 1, 2, 9, 19-22, (2) enzymes [e.g., cyclooxygenase (COX)2, 5-lipoxygenase (LOX), heme oxygenase-1 (HO-1), and inducible nitric oxide synthase (iNOS)] 1, 2, 9, 19, 20, 23, 24, (3) protein kinases [mitogen-activated protein kinases (MAPKs), Janus kinase (JAK), adenosine monophosphate-activated protein kinase (AMPK), protein kinase A (PKA), and PKC 2, 9, 19-22, 24, 25, (4) cell cycle proteins (e.g., cyclin D1 and E) 1, 2, 9, 19-21, 25, (5) cytokines [e.g., tumor necrosis factor (TNF), interleukins (ILs)-1 and 6, and chemokines] 1, 2, 20, (6) receptors [e.g., epidermal growth factor receptor (EGFR) and human EGFR (HER)2)] 2, 9, 19, 20, 23, (7) cell surface adhesion molecules [e.g., intercellular adhesion molecule (ICAM)-1, vascular cell adhesion molecule (VCAM)-1, and endothelial leukocyte adhesion molecule (ELAM)-1 2, 20, and (8) genes that regulate cell proliferation and apoptosis (e.g., p21, p27, and p53) 1, 2, 9, 19-21, 24.

## 3. Osteogenic sarcoma

### 3.1 Osteogenic sarcoma (osteosarcoma)

Primary malignant bone neoplasms are extremely rare malignancies and account for less than 0.2% of all cancers 26. Osteosarcoma, the most prevalent primary bone malignancy, accounts for approximately 35% of all malignant bone tumors 27 and 3-5% of pediatric cancers 26. It shows a peak incidence between 11 and 15 years with an estimated incidence of 3 cases/million population/year and about 6 per million children 28-31 as well as a second peak incidence in older adulthood and 2 per million adults 31. In general, 80-90% of osteosarcoma occurs in long bones, especially the metaphysis, and the femur, tibia, and humerus represent approximately 85% of them 28. Currently, standard clinical procedures for osteosarcoma are preoperative (neoadjuvant) chemotherapy, including cisplatin, doxorubicin (DOX), adriamycin, methotrexate, ifosfamide, and etoposide, *en bloc* or radical excision of the primary bone tumor or amputation of the diseased limb and complex reconstructions, and adjuvant chemotherapy 32.

Prognostic factors for osteosarcoma patients include patient age, tumor site and size, presence and location of metastasis, chemotherapy response, and surgical type and margins 26, 33. Because osteosarcoma cells are not easily killed by radiation, radiotherapy can sometimes be used in patients with positive surgical margins 26, 34. While radiotherapy is administered to try to kill the remaining cancer cells, adjuvant chemotherapy is often followed. Thus, neoadjuvant chemotherapy is routinely used to treat preoperative occult metastases and preserve the diseased limb in more than 90% of osteosarcoma patients 28, 35. However, most treatment failures and high mortality rates result from its metastatic vigor 36, even when aggressively treated with neoadjuvant chemotherapy. If the necrosis of the resected tumor is less than 90% after neoadjuvant treatment, adjuvant chemotherapy regimens can be altered 37.

Generally, four (osteoblastic, fibroblastic, chondroblastic, and telangiectatic) pathologic subtypes of conventional high-grade human osteosarcoma are clinically identified, while no significant differences in clinical outcome correlate with the various subtypes, including the chondroblastic subtype without a favorable response to chemotherapy 29, 31. To date, the long-term survival rate is 60-75% at 5 years for patients with localized tumors 35, but it is drastically reduced to 20-30% if positive pulmonary metastases at diagnosis 38, 39. Potent lung metastasis remains the leading cause of death in osteosarcoma 30, 31. The development of adjuvant therapies to target the molecular basis for abrogating osteosarcoma cell proliferation, invasion, and migration and improving survival rates is crucial 40.

### 3.2 Molecular carcinogenesis, cell death, and metastasis of osteosarcoma

Carcinogenesis (oncogenesis) is a composite process of transforming normal cells into cancer cells in which multiple cell signaling pathways and hundreds of molecules are dysregulated, including biochemical molecules of growth factors and receptors, transcription factors, enzymes, kinases, cytokines, and genes that regulate proliferation, angiogenesis, and apoptosis 41. As a result of activating extrinsic (death receptor) and/or intrinsic (mitochondria) caspases, apoptosis is initiated that manifests as cell shrinkage, plasma membrane bleb, chromatin condensation, and nuclear DNA fragmentation 42-44. In the transmission of the apoptotic pathway, stress-inducible molecules, such as MAPKs)/extracellular signal-regulated protein kinase (ERK), c-Jun N-terminal kinase (JNK), and p38, as well as phosphoinositide 3-kinase (PI3K)/Akt are involved 45-47.

After carcinogenesis, several distinct hallmarks of cancer cells develop, such as evading apoptotic cell death, endless proliferation, and distant metastasis, resulting in host mortality. During tumor growth or disease, a physiological or pathological process, newly formed vascular vessels evolve from pre-existing vessels through angiogenesis 48. Autophagy (autophagocytosis), a cellular degradation process, allows cells to remove unnecessary or dysfunctional components by inducing the lysosomal recycling of intracellular nutrients and caspase-independent cell death in conditions of starvation 49. The orderly degradation and recycling of cellular components also play an imperative role in the homeostasis of non-starved cells. In addition to the promotion of apoptosis 50, JNK and p38 can induce autophagic cell death to regulate autophagy programs 45, 51.

Cancer metastasis involves the dissociation of malignant tumor cells, the epithelial-mesenchymal transition (EMT), intravasation into the circulation, travel through the bloodstream and lymph fluid, survival and evasion of the host immune system, extravasation, mesenchymal-epithelial transition (MET) and adhesion, and colonization and angiogenesis at a distant site 52, 53. Motile cancer cells after EMT degrade the extracellular matrix (ECM) and invade the basement membrane of blood and lymph vessels through a series of discrete steps and various proteases 53. Mesenchymal-derived sarcoma cells are different from epithelial-derived carcinoma cells and are poorly connected to neighboring cells to embed themselves within the ECM. As traveling tumor cells colonize distant sites, the opposite MET is required for metastasis progression. However, the concept of mesenchymal-derived osteosarcoma cells in EMT with the capability to initiate the metastasis cascade has been further applied in the past decade 54-57.

Like other cancer cells, a variety of pathways, including MAPKs, PI3K/Akt, NF-κB, and JAK/STAT3 participate in various signaling cascades of cell motility, adhesion, migration, and invasion in osteosarcoma 47, 56, 58, 59. Identifying molecular insights into the signal transduction pathways involved in carcinogenesis, induction of cell death, and metastasis of osteosarcoma leads to the development of effective treatments available for patients with or without metastasis 30, 31, 53. As a natural product with pleiotropic anticancer activities, curcumin diversifies its inhibitory effects on a multitude of pathways and biochemical cascades involved in tumorigenesis, proliferation and angiogenesis, apoptosis, and metastasis 2.

## 4. Curcuminoids

### 4.1 Structures of curcumin and analogues

Figure 1 shows a summary of the structure of curcumin (bis-α,β-unsaturated β-diketone), which is a 1,3-diketone with methoxy and alcohol groups attached to its two terminal benzene rings, and its natural and synthetic analogs involved in the molecular impact on human osteosarcoma cells 60. The chemical structures of curcumin, DMC, and BDMC are those of a diarylheptanoid with differently substituted aryl rings and a conjugated aliphatic structure. Curcumin is not soluble in water at acidic and neutral pH 8. Due to interactions between acidic alpha hydrogens and basic molecules, the keto-enol structure of curcumin is formatted in the blood, a cardinal keto pattern in acidic and neutral solutions, and a stable enol pattern in alkaline media 61.

### 4.2 Absorption and excretion of curcumin

**Figure 2:**
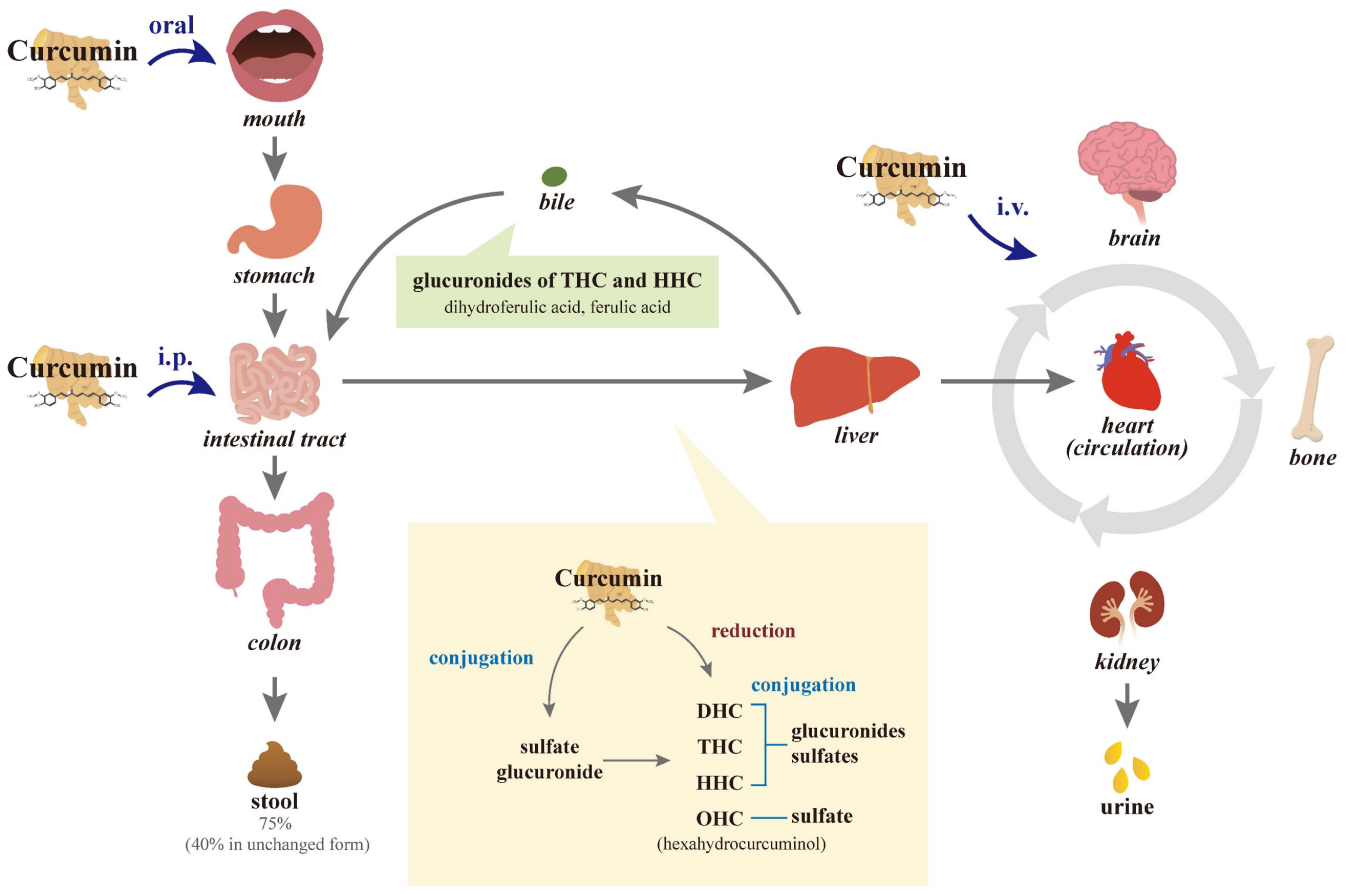
** Diagram of the absorption, metabolism, circulation, and excretion of curcumin in the body.** Due to its poor absorption from the gastrointestinal tract, approximately 75% of the oral intake of curcumin is excreted through the stool passage and negligible amounts are found in the urine. In the intestinal tract and during its first passage through the liver, curcumin is conjugated differently to the glucuronide and sulfate of curcumin in the intestinal tract and liver. Curcumin with or without conjugation is also reduced to dihydrocurcumin (DHC), tetrahydrocurcumin (THC), and hexahydrocurcumin (HHC), and ultimately catalyzed reduction to octahydrocurcumin (OHC), which is alternatively called hexahydrocurcuminol. After first-pass and circulation metabolism, the biliary metabolites are the main glucuronides of THC and HHC and the minor dihydroferulic acid with traces of ferulic acid. Not through intestinal absorption, intraperitoneal (i.p.) injection of curcumin partially subjects to hepatic first-pass elimination, while intravenous (i.v.) injections are directly transported into the circulation.

Because curcumin contains the symmetry of electronegative oxygen groups, the molecule has low solubility: approximately 11 ng/mL in water to be absorbed, approximately 75% of oral intake at a dose of 1 g/kg/rat is excreted through the stool passage, and negligible amounts are found in urine 62, 63, indicating its poor absorption from the gastrointestinal tract (Figure 2). In another study by the same researchers, 10, 80, and 400 mg/rat of curcumin were administered orally, with a constant percentage (approximately 60-66%) of absorption regardless of the administered dose, and approximately 40% of curcumin in an unchanged form could be found in feces over 5 days. However, no detectable amounts of curcumin have been observed in heart blood and urine, and negligible amounts have been observed in the liver (< 5 μg/mL in portal blood) and kidney after 24 h 64, 65. In a human study, administration of a single dose of 2 g of curcumin orally generated a plasma concentration of fewer than 0.01 μg/mL at 1 h and then a gradual decline to zero at 3 h 66, also indicating poor intestinal absorption.

Oral administration of 4-8 g of curcumin at 1-2 h in humans could obtain maximum plasma levels of as little as 0.51-1.77 μM and gradually decrease in 12 hours through avid intestinal sulfation, glucuronidation, and reduction 67. In a human clinical trial, an oral intake of 3.6 g of curcumin at 1 h could produce 11.1 nM of curcumin in plasma 68. After administration of 10 and 12 g of curcumin orally in healthy human volunteers, its glucuronide and sulfate conjugates could be detected mainly in plasma 69, 70, and they appeared to offer low activity, low cell permeability, and a short plasma half-life compared to the free form 68, 71. When administered orally at a dose of 500 mg/kg in rats, 1% bioavailability of curcumin with the parent drug at levels near the detection limit between 5 and 10 nM has been detected in the plasma 72, 73. In another rat study, a low plasma level of 15 ng/mL of curcumin after oral administration of 1 g/kg curcumin for 50 minutes again suggests poor intestinal absorption of curcumin 74.

Not through intestinal absorption, but transport by mesenteric and extra-mesenteric, portal and extra-portal, or lymphatic vessels, intra-peritoneal (i.p.) injection of 0.1 g/kg curcumin to mice at 15 min partially subjected to the elimination of liver first-pass could yield a maximal concentration of 2.25 μg/mL of curcumin in plasma and a maximal amount of curcumin (117 μg/g) in the intestine at 1 h 75. Using intravenous (i.v.) injections directly transported into the circulation, 40 mg/kg of curcumin by i.v. injection in rats decreased in plasma in 1 h 73. After i.v. administration of 10 mg/kg of curcumin in rats, the maximum concentration of unchanged curcumin appeared 0.36 μg/mL in plasma, while, after oral administration of a 50-fold higher dose of curcumin (500 mg/kg), only 0.06 μg/mL of curcumin could be present in serum and the time to reach the maximum concentration was 41.7 min 72.

### 4.3 Metabolism and distribution of curcumin

After absorption, curcumin is subjected to conjugations such as glucuronidation and sulfation, as well as reduction at various tissue sites 76, 77. Several studies in rodents and humans have shown that the main organ responsible for the metabolism of curcumin is the liver 78, 79. In the intestinal tract and during its first passage through the liver of humans and rats, curcumin is biotransformed differently from curcumin glucuronide and curcumin sulfate and it is reduced by different reductases to dihydrocurcumin (DHC), tetrahydrocurcumin (THC), and hexahydrocurcumin (HHC), and ultimately catalyzed reduction to octahydrocurcumin (OHC), which is alternatively called hexahydrocurcuminol 75, 80, 81. The metabolic reaction of curcumin is carried out extensively in hepatocytes and enterocytes, and then the reductive metabolites are easily conjugated 73, 80-83. After first-pass and circulation metabolism, biliary metabolites are the major glucuronides of THC and HHC and the minor dihydroferulic acid with traces of ferulic acid 84.

The metabolic conjugation of curcumin with activated sulfuric or glucuronic acids is more abundant in human intestinal fractions than in rat ones; however, conjugation in human hepatic fractions is less extensive than in rat liver tissues. While curcumin glucuronides, THC, and HHC metabolites, made primarily in the intestines, have their own pleomorphic anticancer, anti-inflammatory, and antioxidant properties, they are less potent than the parent curcumin 80, 85. Conversely, other studies suggested that DMC, curcuminoid glucuronides, and THC are more active than curcumin itself 86-88. Extensive intestinal and liver metabolic biotransformation of conjugation and reduction of curcumin leads to a reduction in unmetabolized curcumin exposed to the general blood circulation and tissues such as bone other than the digestive tract.

When curcumin circulates in the body, quick alterations in its chemical structure through keto-enol tautomerism (enolization) result in low systemic distribution 89. The fast elimination of curcumin from the body is another major cause of its short half-life and low biological activity, especially the intestinal and first-pass metabolism of conjugation and reduction. After i.p. and i.v. injections in rodents, curcumin circulates in the body and large amounts of curcumin and metabolites circulating in the body are excreted in bile; however, major metabolites THC and HHC glucuronides may be reabsorbed into the enterohepatic circulation 64. More than 50% of curcumin is metabolized and secreted into the bile within 2 hours after i.v. administration. Within 72 h after i.p. administration, approximately 73% of curcumin metabolites are excreted in feces and 11% in urine 84; it indicates that their large elimination occurs through bile with relatively little remaining in tissues. Similarly, 6 h after curcumin i.v. injection, approximately 85% of curcumin metabolites have been found in the bile. In addition to traces of the unchanged drug in the liver and kidney, a trace amount (< 5 μg/mL) in the portal blood has been observed from 15 min to 24 h after oral administration of 400 mg of curcumin to rats, while no curcumin could be detected in the heart blood 65.

In rodents, poor diet bioavailability and curcumin levels found in sera are due in part to avid intestinal and first-pass hepatic metabolic biotransformation 61. At an oral dose of 2 g/kg in rats, absorption and elimination half-lives of 0.31 ± 0.07 and 1.7 ± 0.5 h have been reported, respectively, and a maximum concentration of 1.35 ± 0.23 μg/mL in sera has been found at 0.83 h 66. However, at a lower oral dose of curcumin of 1 g/kg in rats, an elimination half-life value of 1.45 h was detected and it showed an insignificant difference from a higher dose of curcumin, indicating the dose independence of the elimination half-life of curcumin in rats 90. The same dose of 2 g/kg of curcumin has shown undetectable or extremely low serum levels (0.006 ± 0.005 μg/mL) at 1 h in humans 66. In another study of rats, the elimination half-life values for oral curcumin (500 mg/kg) and i.v. (10 mg/kg) have been detected to be 28.1 ± 5.6 and 44.5 ± 7.5 h, respectively 72, further suggesting poor absorption and rapid metabolism of oral curcumin.

In a clinical pilot study, colorectal cancer patients with liver metastases who received an oral dose of 0.45 to 3.6 g/day curcumin for 1 week could obtain low nanomolar levels of the parent compound and its conjugates in the portal and peripheral circulation 79. Although trace levels of curcumin products and their metabolic reduction have been detected, no curcumin could be found in liver tissue. Therapeutic doses of curcumin for the liver and other tissues will not be sufficient in humans. In another study, the low bioavailability of oral curcumin has also been observed in patients with advanced colorectal cancer refractory to standard treatments 71. Similarly, other studies have shown the absence of carcinogenesis inhibition of curcumin against lung and breast tumors due to its low bioavailability 10. Therefore, the aqueous insolubility, poor absorption, rapid metabolism, and short half-life of curcumin pessimistically hinder its clinical application.

Curcumin and its metabolites have been interestingly observed to affect the gut microbiota 91, 92. These metabolites influence curcumin biotransformation, producing 23 different metabolites and various metabolic pathways, such as acetylation, demethylation, hydroxylation, and reduction 93, 94. The bidirectional interaction between curcumin and the gut microbiota and *vice versa* suggests the paradox between the poor bioavailability of curcumin and its diverse pharmacological effects. However, curcumin metabolites specifically modulate the microbiota, host intestinal signals, and gut-associated diseases through fine-tuned mechanisms, such as the gut-liver axis and the gut-brain axis 95-101, while in vivo human studies on the subject are still lacking 102.

### 4.4 Toxicity and tolerance of curcumin

Like reports of various antitumor properties of melatonin and zoledronate depending on different cancer types and cell lines specifically 30, 31, 53, several signaling mechanisms with specific cell types and different concentrations have been demonstrated for various effects of curcumin in osteosarcoma and other types of cancer cells 45, 103. Curcumin may exhibit highly pleiotropic effects through multiple cascades serially or in parallel on cells and concentrations of curcumin are dependent. For example, curcumin at a low concentration (up to 25 μM) induces osteoblast apoptosis, whereas osteoblast necrosis has been detected when the curcumin concentration increases to 200 μM 104. However, curcumin at concentrations of 5-25 μM could induce higher cell death in human osteosarcoma MG-63 cells than in human osteoblasts, as malignant cells are much more sensitive to curcumin than their normal cells 19, 105.

The higher curcumin uptake rate in cancer cells than in normal cells partially contributes to the differential effects of curcumin on cancer cells versus their normal counterparts 106. Reduced levels of glutathione in cancer cells that improve curcumin sensitivity may be another factor that contributes to targeted activity 107. Curcumin suppresses cancer cell proliferation and survival by inhibiting NF-κB but not expressing it in normal cells 108. Although higher concentrations of curcumin are hepatocytotoxic, small concentrations can protect hepatocytes 19, 107. Furthermore, colon cancer cells are much more sensitive to low doses of curcumin than human dermal fibroblasts, and there may be a degree of selectivity for neoplastic cells 109. Curcumin induces dose-dependent damage to both the nuclear and mitochondrial genomes, and mitochondrial damage is more extensive in human hepatoma G2 cells 110. Despite the lack of DNA damage at low doses, curcumin at high doses leads to oxidative stress and damaged DNA, which influences carcinogenesis and causes cancer cell death. However, few adverse events due to curcumin and dose-related toxicity could occur, even at a single oral dose of 12 g and up to 12 g per day for 3 months in humans 61, 67.

In addition to the discernible toxicity of mild diarrhea, detectable levels of the parent compound and its conjugates that can inhibit the production of prostaglandin E2 in leukocytes could be generated in plasma and urine after oral doses of 0.5-3.6 g/day curcumin for up to 4 months 68. It recommends a dose of 3.6 g/day orally for evaluation in clinical trials. Owing to the low nonspecific toxicity to normal cells, the protective and therapeutic effects of curcumin against cancers at almost all stages of carcinogenesis, cell proliferation, and metastasis have received significant attention 111. Despite excellent tolerance, the widespread clinical application of curcumin has been severely curtailed because of its imperfect aqueous solubility (11 ng/mL), poor absorption, profligate metabolism, and systemic elimination. Extremely low levels (22-41 ng/mL) of curcumin could be present in the serum even after high amounts (8 g/day) of oral intake, indicating its limited circulation and tissue distribution 69.

### 4.5 Analogues and carriers to improve bioavailability

Poor aqueous solubility, low intestinal absorption, enolization, and fast metabolic biotransformation of curcumin result in an inability to reach required blood and tissue concentrations. In the past decade, chemical approaches to solving this problem have been used by making the molecule more polar, more asymmetrical, and/or adding more electronegative groups. In addition, they have simplified the monocarbonyl structure to increase the solubility of curcumin analogs 89, 112. The lack of alpha hydrogen molecules could prevent enolization in the body, causing a delay in the metabolism and elimination of curcumin analogs.

By conjugating with macromolecules and encapsulating in liposomes, micelles, and nanoformulations, several nanoparticles (NPs)-based delivery systems have emerged to enhance water solubility, and absorption, thus ultimate bioavailability, and targeted delivery to promote anticancer effects in osteosarcoma 113-115. Our state-of-the-art review from the PubMed database of inception dates until January 1, 2023, explored novel insights into curcumin's intracellular and molecular mechanisms, as well as its potential analogs in human osteosarcoma cells *in vitro* and *in vivo*. Additionally, we compiled the available literature on curcumin in numerous formulations targeting human osteosarcoma.

## 5. Curcumin in human osteosarcoma

**Figure 3:**
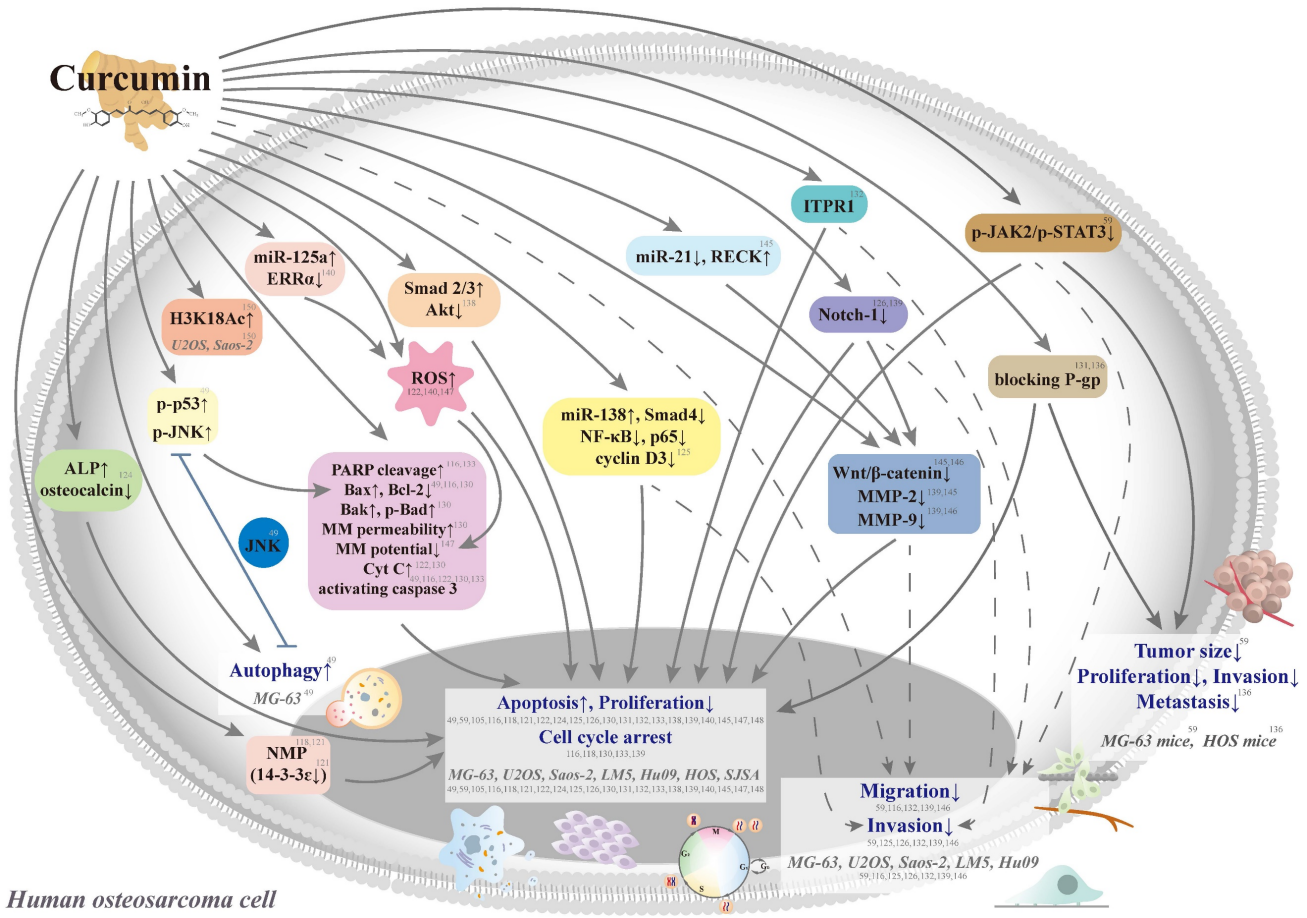
** Schematic representation of various signaling pathways involved in the effects of curcumin on cytotoxicity, antiproliferation, cell cycle arrest, and antimetastasis in human osteosarcoma cells.** The dotted line means that the pathway is controversial because the antimetastatic effect could be induced by the cytotoxic concentrations in the same study. ALP: a*lkaline phosphatase;* Bad: B cell leukemia/lymphoma-2 (Bcl-2)-associated agonist of cell death; Bak: Bcl-2 antagonist killer 1; Bax: Bcl-2-associated X protein; Bcl-2: B cell leukemia-2; Cyt C: cytochrome c; ERR: estrogen-related receptor; H3K18Ac: acetylation of H3K18, a post-translational modification of core histone; ITPR1: inositol 1,4,5‑triphosphate receptor type 1; JAK: Janus kinase; JNK: c-Jun N-terminal kinase; miR: microRNA; MM: mitochondrial membrane; MMP: matrix metalloproteinase; NMP: nuclear matrix protein; PARP: poly(adenosine diphosphate-ribosyl)polymerase; P-gp: P-glycoprotein; RECK: reversion-inducing cysteine-rich protein with *Kazal motifs*; ROS: reactive oxygen species; siRNA: small-interfering RNA; STAT3: signal transducer and activator of transcription 3; Tcf: T-cell factor; and MG-63 WT, MG-63 M8, U2OS, Saos-2, LM5, Hu09 WT, Hu09 m132, HOS, and SJSA: human osteosarcoma cell lines.

Curcumin aims at multiple chemotherapeutic pathways and has the tolerability to act as a potential agent for the primary or adjunct treatment of osteosarcoma 116, 117 (Figure 3). In seven human osteosarcoma cell lines (U2OS, Saos-2, LM5, Hu09 WT, Hu09 m132, MG-63 WT, and MG-63 M8), curcumin has exhibited dose-dependent growth inhibition with half-maximal growth inhibitory concentrations (IC_50_) ranging from 14.4 to 24.6 μM 116 (Table 1). Curcumin could induce apoptosis and G_2_/M phase arrest in seven cell lines through the apoptotic signaling pathways, including cleavage of caspase 3 and poly(adenosine diphosphate-ribosyl)polymerase (PARP), a decrease in cellular levels of the anti-apoptotic protein B cell leukemia/lymphoma-2 (Bcl-2), and an increase in cellular levels of the apoptotic protein Bcl-2-associated X protein (Bax).

The migration activities of Saos-2 and U2OS cells have been half-maximally inhibited with 12.5 μM and 25 μM curcumin, respectively, and part detachment of cells with a rounded appearance and almost complete blockage of migration have been achieved at 50 μM curcumin in the seven cell lines. However, the authors could not speculate on the antimigratory potential wildly, because the cytostatic concentration range of 12.5 μM and 25 μM of curcumin could cause cell death to mislead antimigratory information 116.

### 5.1 Curcumin in MG-63 cells

At the right concentrations of 5-25 μM, curcumin could selectively kill human osteosarcoma MG-63 cells with an apoptotic peak in sub-G_1_ and changes in nuclear matrix proteins (NMPs) over healthy bone cells 105, 118. NMPs are a class of tissue-specific proteins that modulate signal transduction, gene expression, and apoptosis. 14-3-3ɛ, an NMP, can integrate various signaling pathways by interacting with different proteins to regulate the cell cycle and apoptosis 119, 120, and curcumin could suppress the expression level of 14-3-3ɛ and change the co-located apoptosis-associated proteins, including Bcl‑2 and Bax, p53, and c‑Fos transcription factor in MG‑63 cells 121.

Although a low concentration (10 μΜ) of curcumin has reduced the reactive oxygen species (ROS) level in MG-63 cells, a high concentration (80 μΜ) of curcumin could increase the ROS content, the Cyto C release, and the caspase 3 activation 122. Because the ROS scavenger N-acetyl-L-cysteine (NAC) inhibits the release and activation of the apoptosis protein, ultimately hindering curcumin-induced apoptosis, NAC attenuates the toxicity of curcumin, indicating that ROS may contribute to Cyt C release and cell apoptosis in curcumin treatment, indicating curcumin induction of MG-63 cell apoptosis through the ROS/Cyt-C/Caspase-3 pathway.

Curcumin and four potential anticancer agents of aminonaphthoquinone derivatives (Rau 008, 010, 015, and 018) 123, tamoxifen, and 17β-estradiol are working together to treat MG-63 cells, revealing synergistic antiproliferation with increased alkaline phosphatase (ALP) and decreased osteocalcin 124. In MG-63 cells, curcumin inhibits cell proliferation and improves apoptosis and autophagy. By up-regulating the JNK pathway, apoptosis inhibition promotes curcumin-induced autophagy, and autophagy inhibition reversely accelerates curcumin-induced apoptosis 49.

In MG-63 cells, curcumin inhibits proliferation in the microculture tetrazolium colorimetric (MTT) assay and decreases the expression levels of the microRNA (miRNA/miR)-138 target genes Smad4, NF-κB p65, and cyclin D3 125. While overexpression of hsa-miR-138 decreases the Smad4, NF-κB p65, and cyclin D3 expression levels, inhibition of hsa-miR-138 increases them in MG-63 cells, suggesting that curcumin increases the expression of hsa-miR-138 to suppress cell proliferation and invasive capacity by inhibiting its target genes. Regardless, the author's conclusion that 20 μM of curcumin reduced invasive capacity is doubtful, because the curcumin concentration treated at 24 h also showed the potential for cytotoxicity against MG-63 cells (*p* < 0.05), which is mentioned in the study results 125.

Upon exposure to hypoxic conditions, MG‑63 cells have shown increased proliferation and increased invasiveness, and these effects could be prevented with curcumin by inhibiting the up-regulation of Notch 1 induced by hypoxia 126. While overexpression of Notch 1 through Notch 1 cDNA transfection has ameliorated curcumin‑inhibited MG‑63 cell growth under hypoxia, the inhibitory effect on MG‑63 cell growth in hypoxia is demonstrated by suppressing Notch 1 expression. Based on data from pancreatic duct adenocarcinoma and gastric cancer cells under hypoxia that exhibit greater invasiveness and metastasis 127, 128, the authors speculated that curcumin at 5 and 10 μM could inhibit MG-63 cell invasion under hypoxic conditions. However, hypoxic concentrations that could exhibit a cytotoxic effect in the study should be excluded 126.

Increases in apoptosis and accumulation in the G_2_/M phase of MG-63 cells have been demonstrated after curcumin (10 and 20 μM) treatment by suppressing the p-JAK2/p-STAT3 signaling pathway, and 27.6 μM is its half-maximal inhibitory concentration at 24 h 59. The authors concluded that curcumin inhibited migration and invasion using the same concentrations of 10 and 20 μM, which could partially contribute to the cytotoxic effect. Hence, the authors should rule out this possibility. Furthermore, in 80 osteosarcoma patients, the mean expressions of p-JAK2 and STAT3 in primary osteosarcoma tissues with lung metastasis (9.23 and 14.82, respectively) are much higher than those without lung metastasis (2.43 and 5.06, respectively), and a positive correlation is evident between both. In MG-63 xenograft mice, curcumin decreases tumor size and p-JAK2 and STAT3 expressions, further validating tumor growth suppression through the JAK2/STAT3 pathway.

### 5.2 Curcumin in U2OS cells

In U2OS cells, curcumin at a concentration of 50 μM has shown the ability to inhibit nicotine-induced ERK expression 129. Although curcumin and DOX administered together have not shown an additive effect in U2OS cells, additive cytotoxicity with curcumin and cisplatin could be observed 116. Curcumin induces G_1_ phase accumulation and undergoes apoptosis (a sub-G_1_ peak at 6 h and a G_1_ block peak at 12 h) in U2OS cells 130. Through the increase in mitochondrial membrane permeability by down-regulation of Bcl-2 but drastically up-regulating Bax, Bcl-2 antagonist killer 1 (Bak), and phosphorylated Bcl-2-associated agonist of cell death (p-Bad), curcumin triggers the release of cytochrome c (Cyt C) from mitochondria into the cytosol and the activation of downstream caspase 3 and the appearance of apoptosis.

In addition, curcumin improves the cytotoxicity of Adriamycin in multi-drug resistant cells of U2OS/ADM (human osteosarcoma cell line model) and the accumulation of Rhodamine 123, indicating the reverse mechanism of blocking the function of P-gp in the cellular membrane of U2OS/ADM 131. Targeting inositol 1,4,5‑triphosphate receptor type 1 (ITPR1) in curcumin-treated U2OS cells serves an anticancer role by regulating proliferation, apoptosis, migration, and invasion 132. Researchers concluded that 15 μM of curcumin suppressed migration and invasion; however, the cytotoxic effect induced by the same concentration of curcumin should not be neglected.

### 5.3 Curcumin in HOS cells

In HOS cells, curcumin induces phase arrest in G_1_/S and G_2_/M, suppressing cyclin D1, cdc2, and cyclin B1, and activates apoptotic processes through caspase 3 activation and PARP cleavage 133. By encoding the ATP-binding cassette sub-family B member 1 gene, namely the multidrug resistance (MDR)1 gene, which is believed to be the main mechanism of MDR in human osteosarcoma cells, it could overexpress P-glycoprotein (P-gp) 134, 135. Curcumin-mediated reversal of MDR correlates with inhibition of P-gp expression and P-gp efflux pump *in vitro* using MNNG/HOS/MTX cells with different cross-resistance degrees to diamminedichloroplatinum, adriamycin, ifosfamide, and epirubicin, and *in vivo* using a xenograft-nude mouse model 136. Although three mice bearing MNNG/HOS/MTX cells with lung metastasis were detected in the MTX group and no cases were observed in the CUR/MTX group, it is controversial to speculate about the antimetastasis abilities of curcumin before excluding its cytotoxic effect.

Researchers have shown that a mixture of curcuminoid, composed of 77% curcumin, 17% DMC, and 3% BDMC, has enhanced suppression of transforming growth factor (TNF)-stimulated NF-κB in myelogenous leukemia KBM-5 cells and the highest cytotoxicity in pancreatic cancer PANC-1 cells, followed by curcumin, DMC, and BDMC, respectively 112, 137. All curcumin, DMC, and BDMC reduce the viability of U2OS and HOS cells and increase the apoptosis induction of HOS cells by activating Smad 2/3 or inhibiting Akt; moreover, the combined use of them synergistically reduces cell viability and colony formation and induces apoptosis compared to single or two agents 138.

### 5.4 Curcumin in MG-63 and U2OS cells

Curcumin causes significant inhibition of cell growth and G_2_/M phase cell arrest and IC_50_ of curcumin at 72 h are 22.17 ± 1.81, 21.06 ± 1.86, and 22.77 ± 1.70 μM in MG-63, U2OS, and Saos-2 cells, respectively 139. In MG-63 and U2OS cells, curcumin's anticancer activity correlates with the concomitant weakening of Notch 1 and down-regulation of its downstream genes, such as matrix metalloproteinase (MMP)-2 and 9, which are deduced to result in inhibition of cancer cell invasion through Matrigel. Even concentration-dependent inhibition of invasion in MG-63 and U2OS cells by 7.5, 15, and 22.5 μM curcumin for 48 h could not eliminate the cytotoxic effect of 22.5 μM curcumin at 48 h, because this situation appeared to reduce cell viability according to Figure 1 of the study, and the statistical significance of *p < 0.05 should not be ignored. Even so, up-regulation of Notch 1 by complementary DNA (cDNA) transfection rescues curcumin-induced proliferation and invasion inhibition, and down-regulation of Notch 1 by small-interfering RNA (siRNA) conversely potentiates them.

Furthermore, curcumin down-regulates estrogen-related receptor (ERR)α gene expression to induce apoptotic cell death by up-regulating miR-125 in MG-63 and U2OS cells 140. Overexpression of ERRα has reduced curcumin-induced apoptosis and ROS to confer resistance to curcumin in MG-63 and U2OS cells, while silencing of ERRα could sensitize U2OS cells to curcumin, resulting in growth inhibition and up-regulation of cleaved forms of caspase 7 and PARP.

### 5.5 Curcumin in MG-63 and HOS cells

miR-21 is up-regulated in osteosarcoma tissues, and its knockdown decreases the migration and invasion of MG-63 cells 141, 142. On the other hand, miR-21 inhibits apoptosis of Saos-2 cells by targeting caspase 8 143 and can predict poor prognosis in osteosarcoma patients 144. In MG-63 and HOS cells, curcumin up-regulates the reversion-inducing cysteine-rich protein with the Kazal motifs (RECK) protein expression by inhibiting miR-21, and thus subsequently deregulates the canonical Wnt/β-catenin signaling that leads to apoptosis of, indicating that *the* miR-21/RECK/Wnt/β-catenin pathway plays an influential role 145.

### 5.6 Curcumin in U2OS and Saos-2 cells

Curcumin exhibits an antiproliferative effect and suppresses the canonical Wnt/β-catenin pathway by repressing both extrinsic and intrinsic and activated β-catenin/T-cell factor (Tcf) transcriptional activities in U2OS, Saos-2, and HOS cells 146. At a concentration of 5 μM, curcumin improves cytotoxicity, induction of apoptosis, and targeting mitochondria of a C-1 acetoxymethyl analog of 7-deoxypancratistatin, JC-TH-acetate-4 (JCTH-4), selectively in U2OS cells 147. Within the cytotoxic concentration range, 5-20 μM of curcumin also decreased U2OS cell migration and reversed Wnt/β-catenin-enhanced U2OS cell invasion under inhibition of the intrinsic transcriptional activity of β-catenin/Tcf and disruption of nuclear translocation of the β-catenin protein in part by down-regulating MMP-9, a Wnt response gene 146. The authors should, however, exclude the cytotoxic effect before concluding about the antimigratory effect. In Saos-2 cells, curcumin within 6-50 μM could inhibit cell survival and resveratrol and diallyl disulfide enhance cell apoptosis induced by curcumin 148. In U2OS and Saos-2 cells, curcumin could increase H3K18 acetylation (H3K18Ac), which is a post-translational modification of the core histone and commonly correlates with active genes 149, 150.

Overall, the authors concluded curcumin had antimigratory potential under the cytotoxic concentrations of curcumin in the same study 59, 116, 125, 126, 132, 136, 139, 146. Therefore, the clinician should determine whether curcumin has the antimetastatic ability to scale up to a level before applying it meaningfully and practicably.

## 6. Curcumin analogs in human osteosarcoma

**Figure 4:**
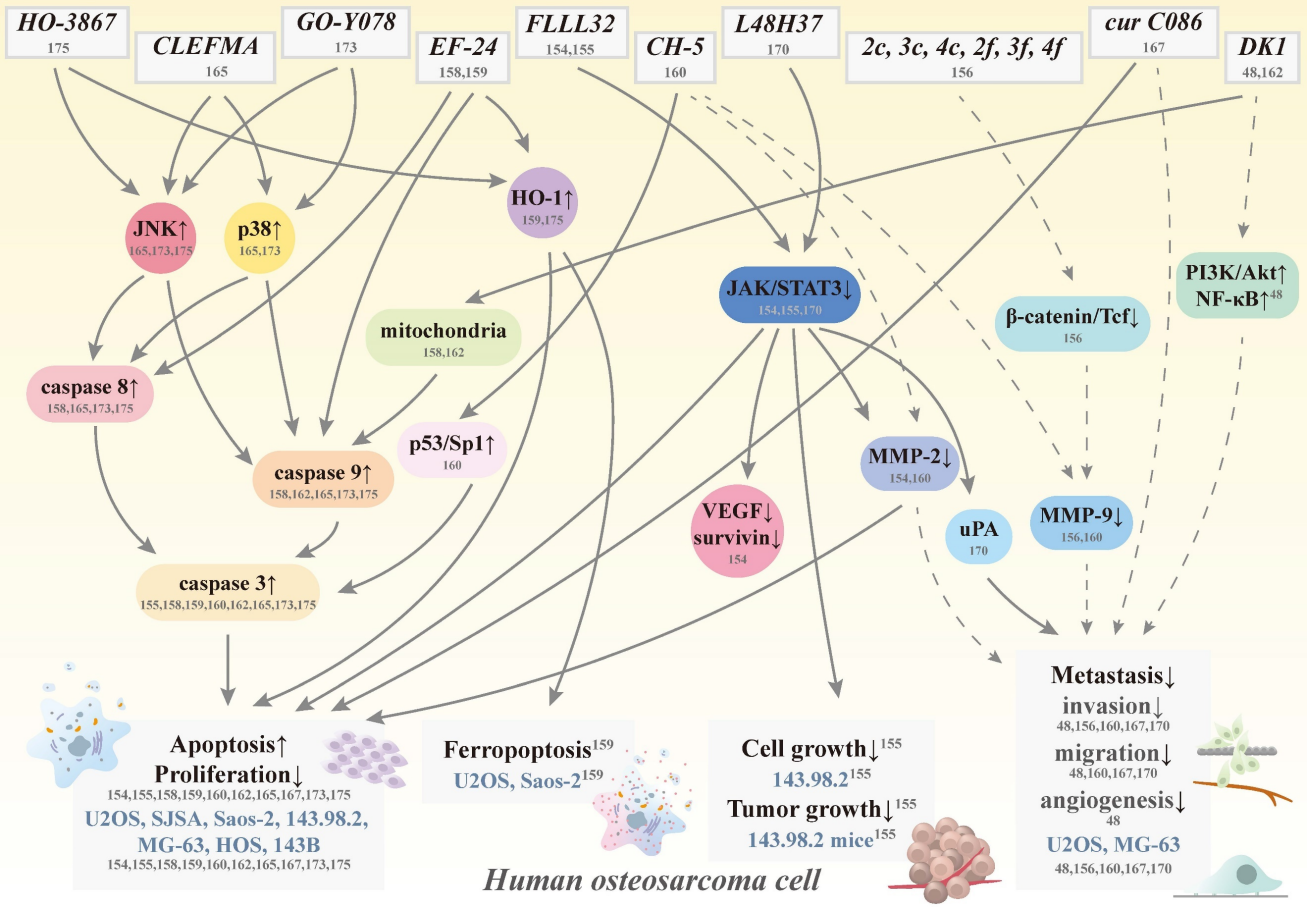
** Schematic representation of various signaling pathways involved in apoptosis, ferropoptosis, antigrowth, and antimetastasis effects of curcumin analogs in human osteosarcoma cells.** The dotted line means that the pathway is controversial because of its cytotoxic effect induced by the same concentration. Cur: curcumin; HO-1: heme oxygenase-1; JAK: Janus kinase; JNK: c-Jun N-terminal kinase; MMP: matrix metalloproteinase; NF-κB: nuclear factor κ-light-chain enhancer of activated B cell; PI3K: phosphoinositide 3-kinase; VEGF: vascular endothelial growth factor; STAT3: signal transducer and activator of transcription 3; Tcf; T-cell factor; uPA: urokinase plasminogen activator; CH-5, CLEFMA, cur C086, DK1, EF-24, FLLL32, GO-Y078, HO-3867, L48H37, 2c, 2f, 3c, 3f, 4c, and 4f: curcumin analogs; and U2OS, SJSA, Saos-2, 143.98.2, HOS, MG-63, and 143B: human osteosarcoma cell lines.

By improving curcumin's solubility, absorption, and stability to increase its bioavailability and potency, this promising natural product will make its way to the forefront of human osteosarcoma therapeutic agents shortly. As such, several curcumin analogs have been further developed to increase the ability to i.v. administration and to prolong half-lives compared to their parent compound to treat osteosarcoma 151, 152 (Figure 4). Through chemical modification to trigger different activities and mechanisms, these anti-osteosarcoma-targeting analogs become more soluble, stable, and potent.

### 6.1 FLLL32 in human osteosarcoma

The diketone analog of curcumin FLLL32, (E)-3-(3,4-dimethoxyphenyl)-1-[1-[(E)-3-(3,4-dimethoxyphenyl)prop2- enoyl] cyclohexyl]prop-2-en-1-one, is a small molecule generated from curcumin using structure-based design and has superior biochemical properties and, more specifically, targets the transcription factor STAT3 for tumor cell proliferation, survival, metastasis, and drug resistance 59, 153. FLLL32 is also more selective in targeting than the parent compound because of two hydrogen atoms replacement on the central carbon of curcumin with a spirocyclohexyl ring 89.

In human osteosarcoma SJSA and U2OS cells, FLLL32 suppresses cell proliferation more than curcumin and enhances proteasome-mediated degradation of STAT3, resulting in a subsequent loss of vascular endothelial growth factor (VEGF), MMP-2, and survivin expressions, and induction of apoptosis 154 (Table 2). Despite inhibiting STAT3 function and expression, FLLL32 decreases STAT3 DNA binding in SJSA cells. Furthermore, the specific inhibitor of JAK2/STAT3, FLLL32, not only decreases cell growth in human osteosarcoma 143.98.2 cells but also delays tumor growth by reducing cell proliferation and inducing cell apoptosis in a mouse model of the 143.98.2 xenograft nude 155. Furthermore, genetic inhibition of STAT3 prevented osteosarcoma 143.98.2 xenograft growth *in vivo*, suggesting that targeting JAK/STAT3 may be a promising therapeutic strategy for osteosarcoma patients.

### 6.2 2c, 2f, 3c, 3f, 4c, and 4f in human osteosarcoma

Curcumin analogs 2c, 2f, 3c, 3f, 4c, and 4f have been synthesized to reduce the β-catenin/Tcf activity to inhibit the Wnt/β-catenin pathway in human osteosarcoma U2OS cells 156. Although 2f, 3c, and 4f have not altered the amount of β-catenin in the cytoplasm of U2OS cells, they could cause a reduction in nuclear β-catenin at 5 μM, indicating a disruption in the translocation of nuclear β-catenin. Reduced U2OS cell invasiveness was also observed at the same concentration (5 μM) and 1 μM by 2f, 3c, and 4f. However, their cytotoxic effects should be excluded before making speculations on the anti-invasive capabilities of U2OS cells to avoid misleading anti-invasiveness by cell death.

### 6.3 EF-24 in human osteosarcoma

To make the structure simple and symmetrical, the designated monoketone analog of curcumin 3,5-bis(2-fluorobenzylidene) piperidin-4-one, also known as EF-24, exhibits broad-spectrum antiproliferative activity in some cancer cell lines 157. The half-life values of the absorption and elimination of EF-24 are 177 and 219 min, respectively, and its bioavailability is 60% and 35% after oral and i.p. administration, respectively. In human osteosarcoma Saos-2 cells, EF-24 has a higher potency than curcumin to inhibit cell growth and to induce cell death, change nuclear morphology, and activate caspases 3 and 7 through the Fas/PARP axis, indicating that EF-24 strongly induces apoptosis through both the death receptor-mediated extrinsic pathway and the mitochondrial-mediated intrinsic pathway 158.

Furthermore, EF-24 induces apoptosis in human osteosarcoma U2OS and Saos-2 cells, which is reversed by ferrostatin-1, but not pan-caspase inhibitor (Z-VAD(Ome)-FMK), dual autophagy kinase ULK1/2 inhibitor (MRT68921), or necrosulfonamide. EF-24 also increases levels of malonydialdehyde (MDA), ROS, and intracellular ferric ions, and these effects are attenuated by ferrostatin-1 159. EF24 up-regulates HO-1 expression and HO-1 overexpression facilitates EF24 to induce ferroptosis in U2OS and Saos-2 cells while silencing of HO-1 attenuates EF24-mediated cytotoxicity and inhibition of glutathione peroxidase 4 (GPX4) expression. These results of up-regulation of HO-1 by EF24 suppressing GPX4 expression and triggering ferroptosis indicate that EF24 may serve as a promising agent for the treatment of patients with HO-1-positive osteosarcoma.

### 6.4 CH-5 in human osteosarcoma

CH-5, a synthetic structural analog of curcumin 4,4'-[(2-Oxo-1,3-cyclohexanediylidene)-di(E)methylylidene] dibenzonitrile, is more potent than curcumin in decreasing cell viability in human osteosarcoma U2OS, MG-63, and Saos-2 cells, and induces apoptosis through increases in caspase 3/7, cleaved PARP-1, and the p53/Sp1 axis in U2OS cells 160. At cytotoxic concentrations of 10-40 μM, CH-5 inhibited migration and invasion as well as the production of MMP-2 and 9; however, the authors may be misled by cell death to conclude that CH-5 is antimetastatic.

### 6.5 DK1 in human osteosarcoma

To improve bioavailability, the synthesized chemical curcumin analog DK1, (Z)-3-hydroxy-1-(2-hydroxyphenyl)-3-phenylprop-2-en-1-one, has been synthesized 161. In addition to cytotoxic activity in breast MCF-7 cancer cells by inducing G_2_/M cell cycle arrest and apoptosis, DK1 leads to more morphological changes in human osteosarcoma U2OS and MG-63 cells and a significant reduction in cell numbers by inducing apoptosis than curcumin, which occurs through the mitochondrial-dependent signaling pathway 162. Furthermore, DK1 has been reported to inhibit metastasis and angiogenesis by up-regulation of PI3K/Akt in U2OS cells and NF-κB in both U2OS and MG-63 cells 48. However, the authors should exclude the effects of IC_25_ and IC_50_ on cell growth before drawing any conclusions about the inhibition of DK1 motility, migration, invasion, and angiogenesis.

### 6.6 CLEFMA in human osteosarcoma

An artificial analog of EF 24, 4-[3,5-bis(2-chlorobenzylidene)-4-oxo-piperidine-1-yl]-4-oxo-2-butenoic acid (named CLEFMA), has various anti-inflammation and anticancer properties in lung adenocarcinoma H441 cells 163, 164. As a potent diphenyldihaloketone analog, CLEFMA not only induces apoptosis of human osteosarcoma U2OS and HOS cells but also triggers activation of the caspase cascade of caspases 8 and 9 initiators and the caspase 3 effector in U2OS cells 165. Through the JNK1/2 and p38 pathways, CLEFMA triggers both intrinsic and extrinsic apoptotic processes in U2OS cells. This suggests that CLEFMA may be a promising agent for the treatment of human osteosarcoma.

### 6.7 Cur C086 in human osteosarcoma

Having been artificially synthesized, the curcumin derivative C086 (cur C086) [1,4,7-tris (4-hydroxy-3-methoxyphenyl)-1,6-heptadiene-3,5-dione], is a structural analog of curcumin and has superior therapeutic effects than natural curcumin, such as aqueous solubility and anticancer properties in colon cancer 166. In human osteosarcoma MG-63 cells, cur C086 down-regulates B lymphoma Mo-MLV insertion region (BMI)1 mRNA and protein expressions and inhibits proliferation dose- and time-dependently 167. Its IC_50_ value for cur C086 is 20 μM and that for cisplatin is 1.28 nM. Although the expression of the polycomb complex protein BMI1 and cell proliferation exposed to cur C086 (20 μM)+cisplatin (1.28 nM) has been decreased, they could synergistically increase the killing effect and induce apoptosis in MG-63 cells. Furthermore, cur C086 treatment increases the chemosensitivity of MG-63 cells to cisplatin or 5-FU. Using the cytotoxic concentration of 20 μM, cur C086 suppresses migration and invasion of MG-63 cells and cur C086+cisplatin (1.28 nM) decreases invasive abilities compared to the control, cur C086, and cisplatin groups. Thus, the authors could not speculate on the antimetastasis effects of cur C086 because cytotoxicity at 20 μM could mislead. Furthermore, proteins P16 and E-cadherin are markedly down-regulated and inhibition of EGFR and Notch 1 is observed in the cur C086+cisplatin treatment group compared to the single treatment group. Therefore, cur C086 is a promising chemotherapeutic agent and cur C086+cisplatin may be a potential chemotherapeutic form for osteosarcoma patients.

### 6.8 L48H37 in human osteosarcoma

Compared to the β-diketone structure of curcumin, the novel curcumin analog L48H37 with an unsaturated monoketone structure1-ethyl-3,5-bis[(*E*)-3,4,5-trimethoxybenzylidene]piperidin-4-one, was manufactured to enhance its bioavailability and antitumor properties in human lung cancer A549 cells and human pancreatic ductal adenocarcinoma PANC-1 and MIA PaCa_2_ cells 168, 169. Up to 5 μM in human osteosarcoma U2OS and MG-63 cells without cytotoxicity, L48H37 suppresses their motility, migration, and invasion 170. L48H37 reduces the protein level, mRNA expression, and promoter activity of the urokinase plasminogen activator (uPA) to interfere with migratory activity in U2OS cells, and also represses the phosphorylation of STAT3, JAK1, JAK2, and JAK3. While STAT3 inhibitor C188-9 decreased uPA expression and migration potential in U2OS cells, STAT3 activator colivelin countered the decrease in uPA expression caused by L48H37, consistent with suppression of invasion and migration by inhibiting uPA expression through the JAK/STAT pathway.

### 6.9 GO-Y078 in human osteosarcoma

By directly interacting with the substrate binding site and its solubility to overcome poor bioavailability, (1E,4E)-1-(4-hydroxy-3,5-dimethoxyphenyl)-5-(3,4,5-trimethoxyphenyl)-penta-1,4-dien-3-one (GO-Y078) was derived from curcumin to enhance inhibition of cell growth 60, 171, 172. GO-Y078 reduces the viability of human osteosarcoma U2OS, MG-63, 143B, and Saos-2 cells in a dose-dependent manner and induces sub-G_1_ fraction cell arrest and apoptosis in U2OS and 143B cells 173. Although GO-Y078 activated both intrinsic caspase 9 and extrinsic caspase 8 initiators to trigger the caspase 3 cascade and PARP in U2OS and 143B cells, suppression of cellular inhibitor of apoptosis (cIAP)-1 and X-chromosome-linked IAP (XIAP) could be detected. In addition to inhibiting IAPs, GO-Y078 simultaneously activates intrinsic and extrinsic apoptotic pathways and cell arrest in U2OS and 143B cells by triggering the JNK and p38 signaling pathways. These results provide a better insight into the mechanisms responsible for the apoptotic properties of GO-Y078 on human osteosarcoma cells and the recognition of GO-Y078 as a promising agent for the treatment of osteosarcoma.

### 6.10 HO-3867 in human osteosarcoma

A novel synthetic curcumin analog HO-3867, 1-[(2,5-dihydro-1-hydroxy-2,2,5,5-tetramethyl-1H-pyrrol-3-yl)methyl]-3,5-bis[(4-fluorophenyl)methylene]-(3E,5E)-4-piperidinone, is a selective and potent STAT inhibitor and an N-hydroxypyrroline derivative of DAPs (diarylidenyl-piperidones), which exhibit targeted cytotoxicity against cancer cells without harming healthy cells 174. HO-3867 reduces the viability of human osteosarcoma U2OS, HOS, and MG-63 cells in a dose-dependent manner and induces apoptosis and sub-G_1_ phase arrest in U2OS and HOS cells 175. In addition to activating effector caspase 3, PARP, and HO-1, HO-3867 triggers the apoptosis cascade of extrinsic caspase 8 and intrinsic caspase 9 initiators in U2OS and HOS cells, while cIAP-1 and XIAP are repressed. Furthermore, the simultaneous induction of both apoptotic pathways in U2OS and HOS cells by HO-3867 is through the JNK signal transduction pathway, contributing to a further understanding of the molecular insight accountable for the apoptotic properties of HO-3867 on human osteosarcoma cells to potentially treat osteosarcoma patients.

## 7. Controlled Formulations for Delivery to Human Osteosarcoma

Due to poor aqueous solubility, insufficient absorption, extravagant metabolism, and hasty systemic clearance, the limited bioavailability of curcumin has hampered its clinical application 60. To harness the anticancer abilities of curcumin, researchers have designed numerous encapsulation-based delivery systems using multifunctional lipid/liposomes, nanoformulations (NPs, nanocarriers, nanoarrays, and hydrogels), micelles, three-dimensional (3D) printed scaffolds, and adjuvants to improve curcumin solubility and allow targeting specific cells and organs of osteosarcoma, ultimately promoting therapeutic effectiveness 113, 176, 177. By applying different materials and techniques to prepare more bioavailable, targeting and penetrating osteosarcoma cells, various formulation methods and delivery systems can be classified as follows:

### 7.1 Lipid nanomaterials

#### Lipid particle

In 11 patients aged 12-60 years (average 18.3) with metastatic high-grade osteosarcoma (7 men and 4 women), high plasma concentrations and non-linear dose dependence could be obtained by oral formulation of solid lipid curcumin particles (SLCPs), and acceptable tolerability has been observed 1 (Table 3). However, the increased bioavailability of curcumin by SLCP was still not defined in the study, as it could be due to increased absorption or reduced conversion of free curcumin.

#### Liposomal NP

The liposome, a globular shape with a central aqueous space and an outer lipid bilayer, varies from 25 to 1,000 nm in diameter 178. It contains one phospholipid bilayer with a hydrophobic tail and a hydrophilic head in the aqueous solution. For drug delivery system targeting, hydrophilic molecules are incorporated into the aqueous interiors of the outstanding weapon of liposomes, while lipophilic particles are incorporated into their hydrocarbon bilayer. Using *NP* or ultrafine particle technology between 1 and 100 nm in diameter, nanoformulations have been developed to resolve the problem of poor bioavailability of the drug. By forming water-soluble complexes through double encapsulation in cyclodextrins (CDs) and followed by liposomes, 2-hydroxypropyl-γ-CD (HPγCD) enhances the water solubility of curcumin from 11 ng/mL to 600 μg/mL, about ~10^4^-fold increase in the aqueous phase of the vesicles 113. When dissolved in dimethylsulfoxide (DMSO), curcumin has been more cytotoxic to osteosarcoma cells at all concentrations investigated 105, 131, 179; however, HPγCD-curcumin liposomal NPs initiate the caspase cascade that results in apoptotic cell death compared to autophagic cell death induced by DMSO-curcumin in human osteosarcoma KHOS cells and KHOS mice 113. The cytotoxic effects of liposomal curcumin on KHOS cells are 2 to 4 times more potent than those detected in non-liposomal formulations.

Because C6 ceramide (C6) is highly insoluble like curcumin, C6 and curcumin have been incorporated into the bilayer of liposomal NP to increase its bioavailability and cytotoxic effects against human osteosarcoma MG-63 and KHOS cells 115. C6-curcumin liposomal NP induces G_2_/M arrest and exhibits combined effects on the expression levels of cyclin D1 and cyclin B1. As liposomal curcumin pegylation has generated 4-5 times more effective than curcumin as a pure compound against osteosarcoma 113, the preparation of curcumin liposomes improves the circulation half-life of curcumin for *in vivo* use 180. As overexpression of the folate (FA) receptor in most cancer cells becomes more malignant, the C6-curcumin-FA liposome represses tumor size and appears to show more apoptosis in KHOS xenograft mice 115.

### 7.2 Polymeric nanomaterials

#### Amphiphilic NP (APNP)

The NP is called amphiphilic because it contains hydrophilic and hydrophobic functional groups. Using a co-dissolution method with acetic acid and dialysis tubes, a homogeneous curcumin solution has been formed in the hydrophobic core of spherical APNP to improve solubility and selectively inhibit the proliferation of human osteosarcoma MG-63 cells 179. Compared to curcumin dissolved in DMSO, the cytotoxicity of curcumin-loaded APNPs in the concentration range of 20-30 μM is more selective for MG-63 cells. Although the viability of MG-63 cells is as low as 15% after treatment with curcumin-loaded APNP at a curcumin concentration of 30 μM, over 50% of human osteoblasts are viable under the same conditions. Therefore, the surfactant-like drug carrier that encapsulates hydrophobic curcumin in its hydrophobic core could selectively inhibit osteosarcoma cells more than healthy osteoblasts.

#### Poly(D,L-lactide-co-glycolic acid) (PLGA) NP

Although water-soluble curcumin-loaded PLGA NP, which improves oral absorption of curcumin, exhibits antiproliferation effects in cisplatin-resistant human oral cancer CAR cells, there is little cytotoxicity for normal human oral keratinocytes and gingival fibroblasts 181. Intriguingly, curcumin-PLGA NP triggers apoptosis of human osteosarcoma U2OS cells through the mitochondria-dependent caspase cascade and the Akt-Bad pathway by up-regulating the expression of cleaved caspases 3 and 9, Bad, cytochrome *c*, and Apaf-1, as well as down-regulating the expression of p-Akt 182.

#### Polymeric NP (PN)

Polymeric NP can circumvent the drawback of poor aqueous solubility of curcumin to improve its systemic circulation. To overcome both dose-limiting side effects and the therapeutic failure incurred by MDR in osteosarcoma, co-encapsulated lipid-coated PN (LPN) of mixed biodegradable polymer core and lipid monolayer shell has been designed to improve inhibitory cell viability of human osteosarcoma KHOS cells 183. Without obvious toxicity, promising dual drugs DOX+Cur co-encapsulated LPNs also enhance antitumor efficiency in KHOS-bearing mice. Accordingly, drug nanotechnology exhibits more curative effects *in vitro* and* in vivo* than its free-drug counterparts. Using spherical polymeric NP as a curcumin carrier (Cur LPN) to optimize curcumin uptake, Cur LPN in HOS cells blocks tumor migration, invasion, and stemness driven by the secretome of acid-stressed mesenchymal stromal cells 184.

In addition to their small size for easy transmission into the cell with less toxicity, such as immunogenicity, and slow degradation and clearance, protein NPs can actively improve curcumin's biodegradability, stability, modification, and control 185. By controlling the particle size and Cur/soy protein NP (SPNP) ratio, a slow-release Cur-containing SPNP (Cur-SPNP) has been designed to increase cytotoxicity, inhibitory activity on cell growth, and intracellular ROS levels in human osteosarcoma Saos-2 cells. Among IC_50_ values of curcumin/SPNP ratios (1:10, 1:20, 1:30, and 1:40), no difference could be detected, whereas all ratios inhibit Saos-2 cell growth. The novel structures for encapsulation and delivery of curcumin increase targeting at the site to improve therapeutic efficiency, as well as to reduce dosage and side effects 186.

Mesoporous silica NPs (MSNs), inorganic-based nanocarriers, characterize a large size with high surface area and large pores to suit high loading capacity and cancer targeting 187. As a ligand molecule, FA may be effectively used to combine with MSNs, loaded with curcumin and a variety of drugs, to target cancer. A phosphonate-functionalized MSN has been fabricated to load colchicine (CL) and curcumin as a core and to bear a polymeric coating of chitosan (CS)-cellulose (CE) conjugated with a coating shell of FA-binding ligand MSNPCLCur/CSCEFA. The constructs MSNPCLCur and MSNPCLCur/CSCEFA improve anticancer activity (increased p53, Bax, and caspase 3, as well as decreased Bcl-2) against human HOS cells.

#### Micelle

A micelle is a colloidal particle formed by the aggregation of amphiphilic molecules dispersed in a liquid. For the delivery of water-insoluble drugs to cancer cells, invertible micellar polymer nanoassemblies (IMAs) have been developed by changing the macromolecular conformation, solubilizing lipophilic drugs in the interiors of IMAs, increasing the polymer concentration, and making local changes to the environmental polarity 188. IMA-delivered curcumin could effectively decrease cell viability in human osteosarcoma MG-63, KHOS, and LM7 cells, but did not influence normal human osteoblasts 189. In MG-63 cells, IMA-delivered curcumin induces G_2_ arrest, contributing to antigrowth and increasing curcumin uptake, thus developing a unique delivery system targeting MG-63 cells.

Owing to overexpression of the CD44 HA receptor and the receptor of hyaluronan (hyaluronic acid, HA)-mediated motility (RHAMM) on the tumor cell surface, tumor cells with high metastatic activity often exhibit increased binding and uptake of HA 190. Alendronate (ALN), a nitrogen-containing bisphosphonate, is currently used to treat bone-related diseases and can be utilized as a bone-targeting ligand 191. A micelle formed by combining hydrophilic HA and hydrophobic octadecanoic acid with ester bonds is stable, and the amphiphilic copolymer HA-octadecanoic acid (HA-C18) has been prepared to be a stable micelle and the curcumin-loaded ALN-HA-C18 micelle exhibits a high affinity for bone and enhances cytotoxic effects on MG-63 cells 176. In mice carrying MG-63, the curcumin-loaded ALN-HA-C18 micelles effectively delay tumor growth, suggesting that ALN-HA-C18 is a potential micelle for delivering hydrophobic curcumin and targeting osteosarcoma.

#### Hydrogel

With the development of MDR, the therapeutic effect of curcumin may be affected, although it is a significant obstacle faced with osteosarcoma chemotherapy 192. β‑CD was adopted to enhance the physicochemical properties of curcumin by incorporating a hydrophobic inner cavity and hydrophilic hydroxyl moieties surrounding the outer surface 193. Compared to natural curcumin in phosphate-buffered saline, a CD-Cur inclusion complex with better solubility and stability has been generated to exhibit significant cytotoxicity efficiency in human osteosarcoma Saos-2 cells 114. Furthermore, the poly(D,L‑lactide‑co‑glycolide)-poly(ethylene‑glycol)‑poly(D,L‑lactide‑co‑glycolide) thermosensitive hydrogel has been selected and synthesized as a novel nanodrug delivery system (NDDS) to co-deliver CD‑Cur and DOX to tumor sites of osteosarcoma. Gel+DOX+CD‑Cur could down-regulate Bcl2, which is closely associated with the prognosis of osteosarcoma patients 194, and up-regulate the protein level of caspase 3, indicating that dual drug nanotechnology improves cytotoxicity consequences and promotes the pro-apoptotic effect of DOX and curcumin bioavailability 114.

Photopolymerization of hydrogels and water-rich 3D structures of target tissue provides mechanical stability and space for tissue regeneration to engineer tumor microenvironments 195, 196. HA is esterified in methacrylate (MA) hydrogel and attracts researchers because of its biological activities and low immunogenicity 197. Silk fibroin (SF), which is extracted from the silkworm (Bombyx mori) cocoon, is widely applied in hydrogel preparation owing to its high biocompatibility, distinctive mechanical characteristics, and a controllable biodegradation rate 198. The development of a HAMA/SF composite hydrogel further enhances its superior mechanical and biocompatible bioactivity to improve tissue reconstruction. For osteosarcoma therapy and bone regeneration, the curcumin-loaded chitosan NP encapsulated SF/HA esterified by MA (CCNPs-SF/HAMA) hydrogel has been developed by treating ethanol and photocuring to provide long-term treatment of osteosarcoma 199. CCNPs-SF/HAMA hydrogel may offer a more sustainable drug release that has an equivalent curcumin concentration of 150 μg/mL for osteoblast proliferation to repair bone defects.

### 7.3 Metal nanomaterials

#### Adjuvant

Because of Ti's excellent potential for biocompatibility, mechanical properties, and corrosion resistance, Ti implants have been increasingly used in recent decades. The plasma spray technique is used to form a homogeneous hydroxyapatite (Ha) coating on the surface of the Ti implant to enhance osseointegration at the tissue-implant interface 200. Due to the stimulation of bone formation and reduction of vitamin K bone resorption 201, the dual-drug delivery of curcumin and vitamin K_2_ (Cur+Vit K_2_) from plasma-sprayed Ha-coated Ti implants enhances osteoblast attachment and proliferation *in vitro*, inhibiting the proliferation of human osteosarcoma MG-63 cells, and *in vivo* osseointegration. The results also highlight the potential of Cur+Vit K_2_-loaded Ha-coated Ti implants for chemoprevention and postoperative defect repair in the treatment of osteosarcoma 202.

### 7.4 Inorganic nanomaterials

#### Nanocarrier

The localized NDDS directly targets cancer cells to enhance the efficacy of the hydrophobic drug, and calcium phosphate (CaP) nanocarriers are an ideal carrier for any local bone treatment 203. Among the CaP nanocarriers, the carbonated apatite (CA) nanocarrier closely mimics natural bone minerals and shows superior suitability as a curcumin carrier compared to the Ha nanocarrier because of its lower crystallinity, increased reactivity, and increased solubility 204. Eggshells mainly comprise calcium carbonate, and eggshell-based apatites exhibit higher levels of curcumin loading and release. The curcumin-loaded eggshell-derived CA nanocarrier exhibits high anti-inflammatory activity and has high cytotoxicity against human osteosarcoma MG-63 cells. These results suggest that eggshell-derived apatite drug nanotechnology is a very suitable way to cure osteosarcoma, prevent post-cancer inflammation, and modulate bone repair and regeneration.

Guided by an external magnetic field, superparamagnetic iron oxide NP (SPION), the most common biocompatible magnetic core, can magnetically control tumor-targeting drug delivery and a switchable hyperthermia system 205. To solve the issue of side effects, chemotherapy resistance, and limited survival ratio, a magnetothermal nanocarrier containing SPIONs coated with a combination formulation of a 3-block copolymer Pluronic F127 and F68 on oleic acid and curcumin-loaded (SPION@OA-F127/F68-Cur) has been designed 206. The nanocarrier SPION@OA-F127/F68-Cur controls the gelation temperature of the shell and the release of curcumin to synergistically exhibit promising bifunctional therapeutic potentials of chemo-*co*-thermal efficacy in killing human osteosarcoma MG-63 cells.

#### Nanoarray

To overcome postoperative osteosarcoma recurrence during bone regeneration, the Ti-based nanoarray device has been utilized for its analogous elasticity to natural bones and has served as a drug vehicle for various therapeutic agents 207. The curcumin-loaded Ti dioxide (TiO_2_) nanoarray has been manufactured using functional CD-based polymer (pCD) coatings and a polydopamine (pDA)-assisted film implemented as the first coating layer on the surface of the TiO_2_ nanoarrays to possess both cancer cell killing ability and excellent bioactivity 208. Curcumin modifies the pCD functionalized TiO_2_/pDA surface to create TiO_2_/pDA/pCD/Cur and promotes apoptosis of human osteosarcoma MG-63 cells by inducing mitochondrial dysfunction caused by ROS overproduction, and, at the same time, inhibits tumor growth of mouse-derived UMR-106 osteosarcoma cell xenograft models. These results successfully demonstrate that the as-prepared TiO_2_/pDA/pCD/Cur construct has anticancer performance and high biocompatibility.

#### Nanofibrous scaffold

Bone scaffolds have a superior potential to repair bone defects after surgical excision by providing better physical support, nutrients and waste transportation, and new tissue ingrowth 209. Encapsulation of curcumin in a liposome to increase bioavailability, followed by incorporation into 3D printed CaP scaffolds with designed porosity, facilitates patient-specific implant fabrication with mechanical interlocking between surrounding host tissue and scaffold 210. Additionally, liposomal curcumin promotes the attachment, proliferation, viability, and differentiation of human fetal osteoblasts. Intriguingly, a Cur-encapsulated liposome-coated 3D printed scaffold with bifunctional bone tissue engineering inhibits human osteosarcoma MG-63 cells and also promotes the formation of healthy bone cells within the porous scaffold, offering a promising drug delivery strategy for the treatment of bone defects after tumor resection 177.

SF is usually used as an implantable fibrous matrix in local drug delivery systems for cancer therapy or defect repair 211. Implantable therapeutic platform pDA-modified curcumin-loaded SF composites have been developed to exhibit excellent bifunctional therapeutic potentials for chemo-*co*-thermal efficacy and bone ingrowth 212. To obtain better mechanical strength, the pDA coating of the biomimetic SF/Cur nanofibrous scaffold significantly represses the cell growth of human osteosarcoma MG-63 cells. Through its excellent photothermal conversion ability and photostability, the biocompatible scaffold with curcumin loading enhances the proliferation of mouse preosteoblast MC3T3-E1 cells. For osteosarcoma, pDA-SF/Cur scaffolds with a curcumin mass ratio of greater than 1% provide a superior, stable, and long-term solution.

## 8. Conclusions and perspectives

**Figure 5:**
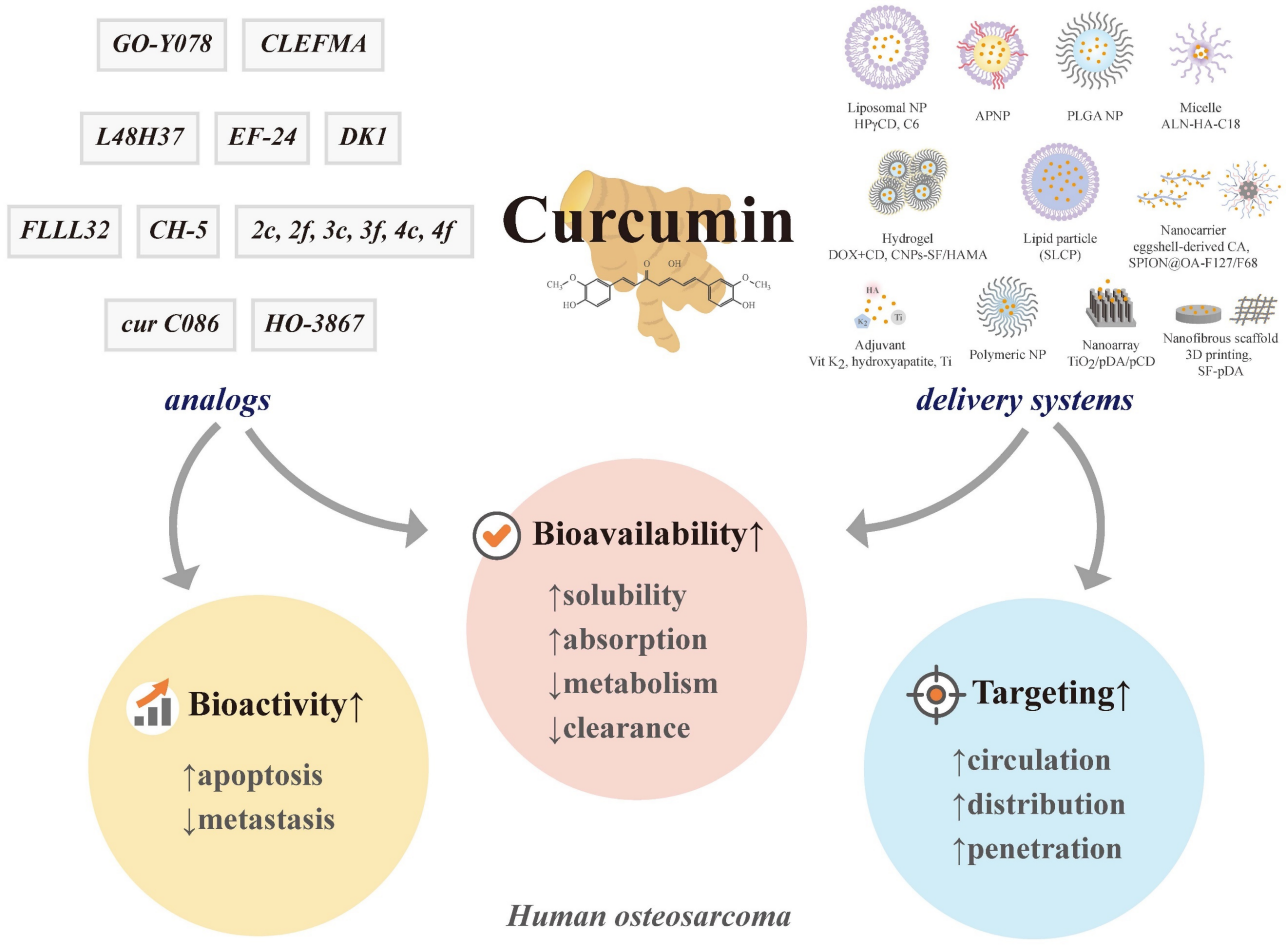
** Current curcumin analogs and carriers to target human osteosarcoma.** ALN-HA-C18: alendronate-hyaluronan-octadecanoic acid; APNP: amphiphilic nanoparticle; CA: carbonated apatite; CNPs-SF/HAMA: chitosan nanoparticles encapsulated silk fibroin/hyaluronic acid esterified by methacrylate; CD: cyclodextrin; C6: C6 ceramide; HA: hyaluronic acid; HPγCD: 2-hydroxypropyl-γ-cyclodextrin; i.v.: intravenous; NP: nanoparticle; pCD: cyclodexin-based polymer; pDA: polydopamine; PLGA: poly(D,L-lactide-co-glycolic acid); SF-pDA: silk fibroin composite coated by polydopamine; SLCP: solid lipid curcumin particle; SPION@OA-F127/F68: superparamagnetic iron oxide nanoparticle coated by a 3-block copolymer Pluronic F127 and F68 on the oleic acid; Ti: titanium; TiO_2_: titanium dioxide; Vit: vitamin; 3D: three-dimensional; CH-5, CLEFMA, cur C086, DK1, EF-24, FLLL32, GO-Y078, HO-3867, L48H37, 2c, 2f, 3c, 3f, 4c, and 4f: curcumin analogs.

To improve poor bioavailability, several current curcumin analogs offer better absorption, stronger potency, slow metabolism, and delayed elimination (Figure 5). To enhance therapeutic efficacy, multifunctional nanoplatforms, such as liposomes, NPs, nanocarriers and nanoarrays, micelles, 3D printing, and other promising novel formulations provide longer periods of circulation, resistance to the metabolic process, and improved targeting of curcumin. However, the existing literature does not yet provide enough evidence to conclude that curcumin in controlled formulations and its analogs are effective in treating human osteosarcoma. Despite combining the advantages of material and design, there is no so-called "most effective" design to co-deliver curcumin and other medicines because the ideal development is in progress; better analogs and carrier systems will be required in the future to achieve better results. In addition to future clinical trials, serious study designs to elucidate and clarify the antimetastatic issue of curcuminoids are warranted to offer more efficient strategies for patients with osteosarcoma.

## Figures and Tables

**Table 1:** Molecular actions of curcumin and curcuminoids on human osteosarcoma in vitro and in vivo

Reference	Mechanism of action	Cell line/*in vivo*	Testing Dose
Huang CY, et al. 2005 129	Inhibits nicotine-induced ERK expression	U2OS	50 μM
Walters DK, et al. 2008 116	Inhibits cell growth and induces apoptosis and G_2_/M arrest by decreasing procaspase 3 and Bcl-2, and increasing PARP cleavage and Bax	MG-63 WT, MG-63 M8, U2OS, Saos-2, LM5, Hu09 WT, Hu09 m132	3.125-50 μM
	Possibly possesses anti-migratory potential		50 μM
	No additive effect with DOX, but additive cytotoxicity with cisplatin	U2OS	15 μM
Jin S, et al. 2009 130	Induces G_1_ arrest and apoptosis via the increase of mitochondrial membrane permeability by down-regulating Bcl-2 and up-regulating Bax, Bak, and p-Bad, then triggering Cyt C release and activating caspase 3	U2OS	5-100 μM
Lee DS, et al. 2009 133	Induces G_1_/S and G_2_/M arrest by down-regulating cyclin D1, cdc2, and cyclin B1, and apoptosis by caspase 3 activation and PARP cleavage	HOS	0.5-20 μg/mL
Leow PC, et al. 2010 146	Inhibits cell proliferation and the Wnt/β-catenin pathway by suppressing both intrinsic and activated β-catenin/Tcf transcriptional activities	U2OS, HOS, Saos-2	1-100 μM
	Seems to inhibit cell migration and invasion under conditions of intrinsic and extrinsic activation of the Wnt/β-catenin pathway by down-regulating MMP-9	U2OS	5-20 μM
Zhao ZL, et al. 2010 118	Induces apoptosis (peak in sub-G_1_) and changes in NMPs	MG-63	7.5 mg/L
Xiao Y, et al. 2011 131	Increases the cytotoxicity of Adriamycin in ADM cells and the accumulation of Rh-123, blocking the function of P-gp	U2OS/ADM	20 μM
Ma D, et al. 2011 147	Enhances cytotoxicity, apoptosis induction, ROS, and mitochondrial targeting induced by JCTH-4 in U2OS	U2OS, Saos-2	5 μM
Masuelli L, et al. 2012 148	Inhibits cell survival Resveratrol and diallyl disulfide enhance Cur-induced apoptosis	Saos-2	6-50 μM
Li Y, et al. 2012 139	Causes cytotoxicity and induces G_2_/M arrest	MG-63, U2OS, Saos-2	7.5-37.5 μM
	Attenuation of Notch 1 and MMP-2 and 9, possibly partial inhibition of invasion	MG-63, U2OS	7.5-22.5 μM
	Up-regulation of Notch 1 by cDNA transfection rescues and down-regulation of Notch 1 by siRNA potentiates Cur-induced proliferation and possibly inhibits invasion	U2OS	15 μM
Si M, et al. 2013 136	Attenuates IC_50_ and RI to multi drugs and inhibits the transport function of P-gp	MNNG/HOS/MTX	30 μM
	Sensitizes antitumor drugs and inhibits proliferation, invasion, and metastasis	MNNG/HOS/MTX BALB/c nude mice (*in vivo*)	5 mg/kg
Collins HM, et al. 2013 150	Increases H3K18Ac	U2OS, Saos-2	10-20 μM
Chang R, et al. 2014 105	Induces greater cytotoxicity than in healthy human osteoblasts	MG-63	5-100 μM
Chang Z, et al. 2014 122	Decreases proliferation, increases ROS, and promotes Cyt C release and caspase 3 activation	MG-63	10-100 μM
	ROS scavenger NAC inhibits the release and activation of the apoptosis protein, ultimately hindering Cur-induced apoptosis		
Yu D, et al. 2015 125	Inhibits cell proliferation by up-regulating miR-138 to down-regulate target genes Smad4, NF-κB p65, and cyclin D3	MG-63	10-40 μM
	Possibly inhibits the invasive ability		20 μM
Pereira MC, et al. 2017 124	Synergistic antiproliferation with increased ALP and reduced osteocalcin combined with Rau 008, 010, 015, and 018, tamoxifen, and 17β-estradiol	MG-63	25-112.5 μM
Wang Z, et al. 2017 126	Inhibits hypoxia-induced proliferation by down-regulating Notch 1	MG-63	5, 10 μM
	Possibly inhibits cells' invasiveness under hypoxia		
Chen P, et al. 2017 140	Inhibits ERRα by up-regulating miR-125a	MG-63, U2OS	10-40 μM
	ERRα confers resistance to Cur-induced apoptosis by scavenging ROS		
Zhang Y, et al. 2017 49	Suppresses proliferation and promotes apoptosis and autophagy	MG-63	0.33-33 μM
	Apoptosis inhibition enhances Cur-induced autophagy via JNK		1-10 μM
	Autophagy inhibition enhances Cur-induced apoptosis and p-JNK		
Lu K, et al. 2018 121	Down-regulates 14-3-3ɛ expression and alters co-located apoptosis-associated proteins	MG-63	7.5 μg/mL
Luo Z, et al. 2018 132	Suppresses proliferation and promotes apoptosis by targeting ITPR1	U2OS	15 μM
	Possibly suppresses migration and invasion by targeting ITPR1		
Sun Y, et al. 2019 59	Inhibits proliferation and induces apoptosis and G_2_/M arrest by inactivating the p-JAK2/p-STAT3 pathway	MG-63	5-80 μM
	Possibly inhibits migration and invasion via p-JAK2/p-STAT3 signaling		10, 20 μM
	Inhibits tumor size and expression of p-JAK2 and STAT3	MG-63 mice (*in vivo*)	50 mg/kg
Zhou L, et al. 2020 145	Inhibits proliferation by decreasing miR-21, increasing RECK, and suppressing Wnt/β-catenin and MMP-2	MG-63, HOS	2.5-10 μM
Huang C, et al. 2020 138	(Cur, DMC, BDMC) decrease cell viability	U2OS, HOS	5-25 μM
	(Cur, DMC, BDMC) induce apoptosis through activation of Smad 2/3 or repression of Akt signaling	HOS	
	(Cur, DMC, BDMC) synergistically reduce colony formation and increase apoptosis		

ADM: multiple-drug resistant of U2OS cell line model; ALP: a*lkaline phosphatase;* Bad: B cell leukemia/lymphoma-2 (Bcl-2)-associated agonist of cell death; Bak: Bcl-2 antagonist killer 1; Bax: Bcl-2-associated X protein; Bcl-2: B cell leukemia-2; BDMC: bisdemethoxycurcumin; cDNA: complementary DNA; Cur: curcumin; Cyt C: cytochrome c; DMC: demethoxycurcumin; DOX: doxorubicin; ERK: extracellular signal-regulated protein kinase; ERR: estrogen-related receptor; H3K18Ac: acetylation of H3K18, a post-translational modification of core histone; IC_50_: half maximal inhibitory concentration; ITPR1: inositol 1,4,5‑triphosphate receptor type 1; JAK: Janus kinase; JCTH-4: JG-TH-acetate-4, a C-1 acetoxymethyl analogue; JNK: c-Jun N-terminal kinase; MDR: multidrug resistance; miR: microRNA; MMP: matrix metalloproteinase; MTX: methotrexate; NAC: N-acetyl-L-cysteine; NF-κB: nuclear factor-κB; NMP: nuclear matrix protein; PARP: poly(adenosine diphosphate-ribosyl)polymerase; P-gp: P-glycoprotein; RECK: reversion-inducing cysteine-rich protein with *Kazal motifs*; RI: resistance index; ROS: reactive oxygen species; siRNA: small-interfering RNA; STAT3: signal transducer and activator of transcription 3; Tcf: T-cell factor; and MG-63 WT, MG-63 M8, U2OS, Saos-2, LM5, Hu09 WT, Hu09 m132, HOS, and SJSA: human osteosarcoma cell lines.

**Table 2:** Molecular actions of curcumin analogs on human osteosarcoma in vitro and in vivo

Reference	Analogue	Mechanism of action	Cell line/*in vivo*	Testing Dose
Fossey SL, et al. 2011 154	FLLL32	Decreases cell proliferation (and Cur)	U2OS, SJSA	0.25-7.5 μM
		Induces apoptosis and proteasome-mediated degradation of STAT3 resulting in decreases in VEGF, MMP2, and survivin		10 μM
		Inhibits STAT3 DNA binding	SJSA	
Leow PC, et al. 2014 156	2f, 3c, 3f, 4c, and 4f	Reduce β-catenin/Tcf activity	U2OS	1 μM
	2f, 3c, and 4f	Disrupt the translocation of β-catenin		5 μM
		Possibly reduce invasion through down-regulation of MMP-9		1, 5μM
	2c	Suppresses β-catenin/Tcf activity		5 μM
Yang SJ, et al. 2014 158	EF-24 (and Cur)	Inhibits cell viability > 3-fold	Saos-2	0.1-100 μM
		Induces apoptosis through the caspase 3/7/8/9 and Fas/PARP axis		
Yan J, et al. 2015 155	FLLL32	Decreases cell growth	143.98.2	1-10,000 nM
		Delays tumor growth and cell proliferation, induces apoptosis, and inhibits genetic STAT3	143.98.2 mice	200 mg/kg
Lima FT, et al. 2018 160	CH-5	Reduces the viability	U2OS, MG-63, Saos-2	5-30 μM
		Induces apoptosis through increases in caspase 3/7 and cleaved PARP-1 and the p53/Sp1 axis	U2OS	10-40 μM
		Possibly inhibits migration and invasion through reduction of MMP-2 and 9		
Aziz MNM, et al. 2018 162	DK1	Inhibits proliferation, alters morphological appearances, and induces accumulation in the S phase and apoptosis via the mitochondria pathway	U2OS, MG-63 (IC_50_: U2OS: 19.6 μM; IC_50_:MG-63: 23.8 μM)	IC_25_-IC_75_
Aziz MNM, et al. 2021 48		Possibly inhibits motility, migration, invasion, and angiogenesis via PI3K/Akt in U2OS and NF-κB in U2OS and MG-63		IC_25_, IC_50_
Yang JS, et al. 2019 165	CLEFMA	Activates extrinsic and intrinsic apoptosis via JNK1/2 and p38	U2OS, HOS	5-80 μM
Jiang X, et al. 2020 167	Cur C086	Decreases BMI1 and proliferation, induces apoptosis, and increases chemosensitivity Possibly suppresses migration and invasion	MG-63	10-80 μM
	Cur C086 + cisplatin	Synergistically induce apoptosis Possibly inhibit migration and invasion Suppress BMI1 expression, P16, E-cadherin, EGFR, and Notch 1		20 μM
Lu KH, et al. 2020 170	L48H37	Represses cellular motility, migration, and invasion	U2OS, MG-63	1.25-5 μM
		Suppresses cell migration by inhibiting uPA via JAK/STAT signaling	U2OS	
Lin H, et al. 2021 159	EF-24	Induces cell death, reversed by ferrostatin-1	U2OS, Saos-2	0.5-4 μM
		Increased MDA, ROS, and intracellular ferric ion levels, attenuated by ferrostatin-1		0.75, 1.5 μM
		Up-regulates HO-1, regulated by ferrostatin-1		0.5-2 μM
		HO-1 overexpression facilitates EF24 to induce ferroptosis; HO-1 knockdown attenuates EF24-induced cytotoxicity and inhibition of GPX4		0.5, 1 μM
Lu PWA, et al. 2021 173	GO-Y078	Decreases cell viability	U2OS, MG-63, 143B, Saos-2	1-16 μM
		Induces both apoptotic pathways by activating JNK and p38 and repressing IAPs	U2OS, 143B	1-8 μM
Lu PWA, et al. 2022 175	HO-3867	Decreases cell viability	U2OS, HOS, MG-63	2-32 μM
		Induces sub-G1 arrest and both apoptotic pathways via JNK signaling	U2OS, HOS	2-16 μM

BMI1: B lymphoma Mo-MLV insertion region 1; Cur: curcumin; EGFR: epidermal growth factor receptor; GPX4: glutathione peroxidase 4; HO-1: heme oxygenase-1; IAP: inhibitor of apoptosis; JAK: Janus kinase; JNK: c-Jun N-terminal kinase; MDA: malonydialdehyde; MMP: matrix metalloproteinase; NF-κB: nuclear factor κ-light-chain enhancer of activated B cell; PARP: poly(adenosine diphosphate-ribosyl)polymerase; PI3K: phosphoinositide 3-kinase; ROS: reactive oxygen species; STAT3: signal transducer and activator of transcription 3; Tcf: T-cell factor; uPA: urokinase plasminogen activator; VEGF: vascular endothelial growth factor; C086, CH-5, CLEFMA, DK1, EF-24 (diphenyldifluoroketone), FLLL32, GO-Y078, HO-3867, L48H37, 2c, 3c, 4c, 2f, 3f, and 4f: curcumin analogues; and U2OS, SJSA, Saos-2, 143.98.2, HOS, MG-63, and 143B: human osteosarcoma cell lines.

**Table 3:** Effects of controlled formulations for the delivery of curcumin to human osteosarcoma in vitro and in vivo

Reference	Delivery system	Mechanism of action	Cell line/*in vivo*	Testing Dose
Gota VS, et al. 2010 1	SLCP	Increases bioavailability and improves tolerability	11 patients with metastatic OS	2000-4000 mg
Dhule SS, et al. 2012 113	HPγCD-Cur liposomal NP	Induces apoptosis and autophagy	KHOS	5-30 μg/mL
		Induces apoptosis	KHOS mice	1.25-10 μg/mL
Dhule SS, et al. 2014 115	C6-Cur liposomal NP	Enhances 1.5 times the cytotoxic effect and induces G_2_/M arrest and shows a combined effect on cyclins D1 and B1	MG-63, KHOS	4-28 μg/mL
	C6-Cur-folate liposomal NP	Reduces tumor size and increases apoptotic cells	KHOS mice	4 μg
Peng SF, et al. 2014 182	Cur loaded PLGA NP	Causes apoptosis via mitochondria-dependent and Akt-Bad signaling	U2OS	0.25-2 μg/mL
Chang R, et al. 2015 179	Cur-loaded APNP	Selective cytotoxicity	MG-63	3-30 μM
Kudina O, et al. 2015 189	IMA	Decreases cell viability	MG-63, KHOS, LM7	1-40 μM
		Induces G_2_ arrest	MG-63	20 μM
		Increases uptake of Cur	MG-63, KHOS, LM7	
Wang L, et al. 2016 183	DOX+Cur LPN	More inhibitory effects on cell viability	KHOS	0.5-50 μM
		More obvious tumor regressions	KHOS mice	10, 20 mg/kg
Xi Y, et al. 2019 176	Cur-loaded ALN-HA-C18 micelle	Exhibits high affinity for bone and high cytotoxic activity	MG-63	1.25-40 μM
		Delays tumor growth	MG-63 mice	25 mg/kg
Verma AH, et al. 2019 204	Cur releasing eggshell derived CA nanocarrier	High anti-inflammatory and cytotoxic activity	MG-63	5 mg/mL
Zhang M, et al. 2019 208	TiO_2_/pDA/pCD/Cur	Promotes apoptosis by activating ROS-induced mitochondrial dysfunction	MG-63	0.2-1.6 mg/mL
Sarkar N, et al. 2019 177	Cur-encapsulated liposome-coated 3D printed scaffold	Promotes cell attachment, proliferation, viability, and differentiation	MG-63	Encapsulation: 44-68% Seeding: 61.19±1.43%
		Inhibits bone cancer cells and promotes the formation of healthy bone cells within the porous scaffold		
Sarkar N, et al. 2020 202	Cur+Vit K_2_-loaded Ha-coated Ti implant	Lower cell proliferation	MG-63	25 μg
Yang Z, et al. 2020 114	Gel+DOX+CD‑Cur	Down-regulates Bcl-2 and up-regulates caspase 3	Saos-2	1 mg/mL
Di Pompo G, et al. 2021 184	Cur LPN	Blocks tumor stemness, migration, and invasion	HOS	4 μM
Yu Q, et al. 2021 199	CCNPs-SF/HAMA	90-400 μg/mL: decreases cell survival 150 μg/mL: has anticancer activity and promotes osteoblast proliferation	MG-63	50-400 μg/mL
Meng Z, et al. 2021 212	SF/Cur-pDA	Exhibits a dose- and time-dependent inhibition effect on cell growth by synergetic chemo-photothermal therapy	MG-63	0.05-2%
Khodaei A, et al. 2021 206	SPION@OA-F127/F68-Cur	A synergistic and non-intensive approach of mild hyperthermia (41°C as the optimum temperature) and curcumin release to induce apoptosis	MG-63	47-61 μM
Zare-Zardini H, et al. 2022 186	Cur-SPNP	Increases cytotoxicity, inhibition of cell growth, and intracellular ROS All ratios inhibit cell growth, with no difference in IC_50_ between Cur/SPNPs ratios.	Saos-2	5 mg/mL (Cur/SPNPs: 1/10, 1/20, 1/30, and 1/40)
AbouAitah K, et al. 2022 187	MSNPCLCur/CSCEFA	MSNPCLCur and MSNPCLCur/CSCEFA improve anticancer activity (increased p53, Bax, and caspase 3; decreased Bcl-2)	HOS	up to 100 μg/mL

ALN-HA-C18: alendronate-hyaluronan-octadecanoic acid; APNP: amphiphilic nanoparticle; Bcl-2: B cell leukemia/lymphoma-2; CA: carbonated apatite; CCNPs-SF/HAMA: curcumin-loaded chitosan nanoparticles encapsulated silk fibroin/hyaluronic acid esterified by methacrylate; CD: cyclodextrin; Cur: curcumin; C6: C6 ceramide; DOX: doxorubicin; HA: hyaluronan, hyaluronic acid; Ha: hydroxyapatite; HPγCD: 2-hydroxypropyl-γ-cyclodextrin; IMA: invertible micellar polymer nanoassembly; LPN: lipid-coated polymeric nanoparticle; MMP: matrix metalloproteinase; NP: nanoparticle; OS: osteosarcoma; pCD: cyclodexin-based polymer; pDA: polydopamine; PLGA: poly(D,L-lactide-co-glycolic acid); SLCP: solid lipid curcumin particle; SPION@OA-F127/F68-Cur: superparamagnetic iron oxide nanoparticle coated by a 3-block copolymer Pluronic F127and F68 on the oleic acid and curcumin-loaded; SF/Cur-pDA: curcumin-loaded silk fibroin composite coated by polydopamine; Ti: titanium; TiO2: titanium dioxide; Vit: vitamin; 3D: three-dimensional; and KHOS, U2OS, MG-63, LM7, Saos-2, and HOS: human osteosarcoma cell lines.

## Abbreviations

ADM: multiple-drug resistant of U2OS cell line model
ALN-HA-C18: alendronate-hyaluronan-octadecanoic acid
ALP: alkaline phosphatase
AP: activator protein
APNP: amphiphilic nanoparticle
Bad: B cell leukemia/lymphoma-2 (Bcl-2)-associated agonist of cell death
Bak: Bcl-2 antagonist killer 1
Bax: Bcl-2-associated X protein
Bcl-2: B cell leukemia/lymphoma-2
BDMC: bisdemethoxycurcumin
BMI1: B lymphoma Mo-MLV insertion region 1
CA: carbonated apatite
CCNPs-SF/HAMA: curcumin loaded chitosan NPs encapsulated silk fibroin/hyaluronic acid esterified by methacrylate
CD: cyclodextrin
COX: cyclooxygenase
Cur: curcumin
Cyt C: cytochrome c
C6: C6 ceramide
cDNA: complementary DNA
cIAP: cellular inhibitor of apoptosis
DHC: dihydrocurcumin
DMC: demethoxycurcumin
DOX: doxorubicin
ECM: extracellular matrix
EC_50_: 50% of maximal effect
EGFR: epidermal growth factor receptor
EMT: epithelial-mesenchymal transition
ERK: extracellular signal-regulated protein kinase
ERR: estrogen-related receptor
GPX4: glutathione peroxidase 4
HA: hyaluronan, hyaluronic acid
Ha: hydroxyapatite
HER: human epidermal growth factor receptor
HHC: hexahydrocurcumin
HO-1: heme oxygenase-1
HPγCD: 2-hydroxypropyl-γ-cyclodextrin
H3K18Ac: acetylation of H3K18, a post-translational modification of core histone
IAP: inhibitor of apoptosis
IC_50_: half maximal inhibitory concentration
IL: interleukin
IMA: invertible micellar polymer nanoassemblie
ITPR1: inositol 1,4,5‑triphosphate receptor type 1
iNOS: inducible nitric oxide synthase
JAK: Janus kinase
JCTH-4: JG-TH-acetate-4, a C-1 acetoxymethyl analogue
JNK: c-Jun N-terminal kinase
LOX: lipoxygenase
LPN: lipid-coated polymeric nanoparticle
MAPK: mitogen-activated protein kinase
MDA: malonydialdehyde
MDR: multidrug resistance
MET: mesenchymal-epithelial transition
miR: microRNA
MMP: matrix metalloproteinase
mmp: mitochondrial membrane permeability
MTT: microculture tetrazolium colorimetric
MTX: methotrexate
NAC: N-acetyl-L-cysteine
NF-κB: nuclear factor κ-light-chain enhancer of activated B cell
NMP: nuclear matrix protein
NP: nanoparticle
OHC: octahydrocurcumin
OS: osteosarcoma
PARP: poly(adenosine diphosphate-ribosyl)polymerase
pCD: cyclodexin-based polymer
pDA: polydopamine
PI3K: phosphoinositide 3-kinase
PLGA: poly(D,L-lactide-co-glycolic acid)
P-gp: P-glycoprotein
PPAR: peroxisome proliferator-activated receptor
RECK: reversion-inducing cysteine-rich protein with Kazal motifs
RI: resistance index
ROS: reactive oxygen species
SF-pDA: silk fibroin composite coated by polydopamine
siRNA: small-interfering RNA
SLCP: solid lipid curcumin particle
STAT: signal transducer and activator of transcription
Tcf: T-cell factor
THC: tetrahydrocurcumin
Ti: titanium
TiO2: titanium dioxide
uPA: urokinase plasminogen activator
VEGF: vascular endothelial growth factor
Vit: vitamin
3D: three-dimensional
